# Targeting the HIF1A-UCA1-PTBP3 axis: a potential therapeutic strategy for head and neck cancer

**DOI:** 10.1186/s12885-025-15020-z

**Published:** 2025-10-09

**Authors:** Lydia Chin-Ling Sim, Yi-Zih Kuo, Tsun-Chih Cheng, Chih-Ling Chen, Jenn-Ren Hsiao, Mei-Xuan Ooi, Zixi Yun, Hung-Ying Kao, Sen-Tien Tsai, Li-Wha Wu

**Affiliations:** 1 Institute of Molecular Medicine, College of Medicine, National Cheng Kung University, Tainan, Taiwan, R.O.C.; 2 Department of Otolaryngology, National Cheng Kung University Hospital, College of Medicine, National Cheng Kung University, Tainan, Taiwan, R.O.C.; 3 Department of Otolaryngology, An Nan Hospital, China Medical University, Tainan, Taiwan, R.O.C.; 4 Department of Biochemistry, School of Medicine, Case Western Reserve University, Cleveland, OH U.S.A.; 5 Research Department, Changhua Christian Hospital, Changhua City, Taiwan, R.O.C.; 6 Department of Laboratory Science and Technology, College of Health Sciences,, Kaohsiung Medical University, Kaohsiung, Taiwan, R.O.C.

**Keywords:** Head and neck cancer, Hypopharyngeal cancer, LncRNA, UCA1, PTBP3

## Abstract

**Background:**

Long non-coding RNAs (lncRNAs) are multifunctional regulators of gene expression and play a vital role in various physiological and pathological processes. The urothelial cancer-associated 1 (UCA1) lncRNA is involved in the development and progression of bladder, colon, stomach, lung, and breast cancers. Pharyngeal cancer, especially those in the hypopharynx (HPC), is a devastating subtype of head and neck cancer often detected in late stages. Although some patients survive under curative treatments, they are still prone to having the disease relapse or metastatic spread. Upregulation of UCA1 expression is associated with disease progression and poor prognosis in HPC patients, but the molecular mechanism by which UCA1 functions in HPC remains unclear.

**Methods:**

We used genetic silencing and ectopic expression approaches to examine the effect of UCA1 dysregulation on the proliferation, colony formation, migration, and invasion of head and neck cancer cells in vitro. Using a xenograft animal model, we employed UCA1-depleted cells to evaluate UCA1’s role in tumor growth and metastasis. RNA pulldown followed by mass spectrometry was used to identify UCA1-interacting proteins. We also examined the downstream effectors and upstream mediator(s) of the UCA1-mediated signaling axis by using RT-qPCR, Western blotting, promoter-driven luciferase, and transendothelial migration assays.

**Results:**

Ectopic UCA1 expression increased cell migration and invasion but reduced the proliferation of head and neck cancer cells. The increases in migration and invasion are accompanied by elevated levels of epithelial and mesenchymal markers, reminiscent of incomplete epithelial-mesenchymal transition (EMT). In contrast, depletion of UCA1 had opposite effects. Animal xenograft studies also supported the role of UCA1 in promoting lymph node metastasis. UCA1 was predominantly nuclear localized, where polypyrimidine tract binding protein 3 (PTBP3) binds UCA1 lncRNA. Manipulating PTBP3 expression reversed the effect of UCA1 expression on cell migration and invasion. Moreover, increased UCA1 expression, mimicking the regulatory effect of TGF-β on cyclin D1, p21, and Smad2 phosphorylation, could promote trans-endothelial migration. Significantly, hypoxia induced UCA1 expression partly through an HIF1A-dependent manner.

**Conclusions:**

We conclude that HIF1A-induced nuclear UCA1 stimulated cell migration and invasion via interacting with PTBP3, increasing lymph node metastasis in head and neck cancer.

**Supplementary Information:**

The online version contains supplementary material available at 10.1186/s12885-025-15020-z.

## Introduction

Head and neck cancers, including pharyngeal cancers, represent heterogeneous diseases, and aggressive tumors in these areas significantly contribute to high morbidity, mortality, and recurrence [1]. Early diagnosis and treatment are thus necessary for minimizing their morbidity and mortality. Hypopharyngeal cancer (HPC) is the most severe subtype with the poorest outcome among pharyngeal cancers [2, 3]. Aging, cigarette smoking, betel nut chewing, and excessive consumption of alcohol are major risk factors [4]. Squamous cell carcinoma is the most common histology and accounts for more than 90% of head and neck cancers [5]. These cancer cells can spread into the blood or lymphatic system [6], and most head and neck cancer patients are diagnosed at the advanced stage [7], Making them difficult to treat. Despite advances in both diagnosis and treatment, over 65% of head and neck cancer patients experience recurrence, metastatic disease, or both [8]. Thus, a non-invasive biomarker is crucial for early detection of head and neck cancer, especially HPC, while preserving the function of the pharynx and adjacent organs [9].

Long non-coding RNAs (LncRNAs) with > 200 nucleotides in size have been detected in blood circulation and shown great promise as cancer biomarkers [10]. Urothelial carcinoma-associated 1 (*UCA1*) is an lncRNA that plays a key role in tumorigenesis [11]. A higher *UCA1* level has been found in head and neck cancer tissues [12] than in their normal counterparts. *UCA1* overexpression increases cell proliferation and promotes resistance to cisplatin, a standard chemotherapeutic drug used in treating oral squamous cell carcinoma [13]. However, the mechanism responsible for *UCA1* dysregulation and its exact mechanisms underlying *UCA1*-mediated tumorigenesis in hypopharyngeal cancer remains unclear.

The *UCA1* gene has three exons and generates two spliced isoforms with sizes of 1.4 and 2.2 kb [14, 15]. The shorter isoform was originally identified as a diagnostic marker for bladder cancer [14] and proposed to function as a molecular sponge that absorbs tumor-suppressive miRNAs in bladder cancer and glioma [16, 17]. The longer isoform, also known as *c*ancer *u*pregulated *d*rug *r*esistant (CUDR), also promotes cancer stemness and drug resistance [18, 19]. Although both *UCA1* isoforms have been studied individually, it remains to be seen whether they function similarly or are produced in the same manner within the same cellular contexts.

In addition to acting as miR sponges, lncRNAs can regulate gene expression through interactions with proteins, thereby influencing transcription, post-transcriptional processing, or mRNA translation [20]. In gastric cancer, *UCA1* enhances CCND1 expression by interacting with EZH2 [21]. Notably, the various functions of lncRNAs significantly rely on their subcellular locations, with some found in extracellular vesicles and others in the cytosol or nucleus [20]. Therefore, identifying their subcellular localization and interacting partners of lncRNAs is critical for elucidating mechanisms by which lncRNAs regulate cellular functions and cancer progression.

Although increased levels of *UCA1* are associated with reduced clinical outcomes in HPC [22], the detailed mechanism in which *UCA1* contributes to the development and progression of HPC is not completely understood. In this study, we identified a new mechanism where hypoxia-inducible factor 1 A (HIF1A) triggers the production of UCA1. Additionally, we discovered a protein associated with UCA1, called polypyrimidine tract binding protein 3 (PTBP3), which plays a role in head and neck cancers.

## Materials and methods

### Cell culture

FaDu line (BCRC 60214), derived from hypopharyngeal squamous cell carcinoma, was grown in minimum essential media (MEM) with 10% fetal bovine serum (FBS), 0.1 mM non-essential amino acid (NEAA), 1 mM sodium pyruvate, and 100 units/ml penicillin and streptomycin. Detroit 562 cells (BCRC 60119) from pharyngeal cancer were Maintained in MEM plus 10% FBS, 2mM L-glutamine, 0.1 mM NEAA, 1 mM sodium pyruvate, 0.1% lactalbumin hydrolysate, and 100 units/ml penicillin and streptomycin. Both lines were purchased from the Bioresource Collection and Research Center (BCRC, Hsinchu, Taiwan) and have been authenticated using STR profiling within the last three years. Other head and neck cancer cells were maintained and propagated as previously described [23]. Immortalized human normal oral keratinocytes (HNOK) from Applied Biological Materials Inc. were a generous gift from Dr. Huang TT at National Cheng Kung University. Human 293T cells (CRL-3216) were maintained as described by the American Tissue Culture Collection (ATCC, USA). Human immortalized microvascular endothelial cell line HMEC-1 [24] was grown in Endothelial Cell Growth Medium-2 (Lonza Bioscience, Walkersville, Maryland, USA). All cell lines were incubated at 37 °C in a 5% CO2 incubator. All experiments were performed with mycoplasma-free cells.

### RNA interference

Detroit 562 was transfected for 24–48 h with control siRNA (si-Ctrl) or UCA1 siRNA (si-UCA1) SMARTpool purchased from GE Healthcare Dharmacon (Lafayette, CO, USA) prior to RNA isolation or cell-based functional assay. The *UCA1* siRNA SMARTpool targets all isoforms. The target sequences include UGUUAGAGGGCUUGGGACA (N-188002-01), CACCCUAGCUGGACGAUCA (N-188002-02), ACCCUAGACCCGAAACUUA (N-188002-03) and GAUUAGGCCGAGAGCCGAU (N-188002-04).

### Subcellular RNA fractionation

Nuclear and cytoplasmic RNAs were isolated based on minor modifications of the published protocols [25]. Briefly, the indicated cells were collected and lysed in a 10 mM Tris buffer containing 140 mM NaCl, 1.5 mM MgCl2, 0.5% CA-630, and RNase inhibitor. After centrifugation, supernatants were collected as cytoplasmic fractions. Following extensive washing, the nuclear pellet was either lysed in an SDS lysis buffer for Western blot analysis or total RNA isolation. Following respective validation with lamin B (a nuclear marker) and GAPDH (a cytosolic marker) by Western blot analysis, we isolated total RNA from either fraction using TRIzol Reagent for RT-qPCR.

### In situ hybridization (ISH) 

Following seeding the indicated cells on the Lab-Tek II Chamber Slide™ (Thermo Fisher Scientific, Waltham, MA, USA), pre-coated overnight with poly-L-lysine, we fixed them for 30 min in 10% neutral buffered formalin. They were then pre-treated for 10 min with a diluted protease III. ISH assay for UCA1 expression was performed by using RNAscope™ Probe- Hs-UCA1 (Table S1) as described by the manufacturer (Advanced Cell Diagnostics, Newark, CA, USA). The colorimetric signals were photographed under light microscopy.

#### Murine tumorigenesis and metastatic spread

Male NOD/SCID mice at 6 weeks old were ordered from and housed in a standard pathogen-free facility at the Laboratory Animal Center at National Cheng Kung University. The use of these animals and experimental protocols was reviewed and approved by the Institutional Animal Care and Use Committee (IACUC) at NCKU (IACUC#111139). To sustain the depletion of *UCA1*, we used CRISPR interference (CRISPRi) [26] to reduce endogenous *UCA1* expression in Detroit 562 cells and validated *UCA1* reduction in these cells by RT-qPCR (Fig. S2A). Following the intraperitoneal injection of an anaesthetic cocktail containing Zoletil (50–60 mg/kg) and Xylazine (2.3–2.7 mg/kg) and confirmation of adequate anaesthesia, we suspended control (sgCtrl) or *UCA1*-depleted cells (sg*UCA1* clone 2) in phosphate-based saline and, respectively, injected 1 × 10^5^ manipulated cells/50 µl into the buccal mucosa of two mouse groups, sgCtrl and sg*UCA1* (*N* = 4 in each group). For ectopic *UCA1* expression, we randomized the mice into two groups: Vector and *UCA1* (*N* = 7 per group). Their body weights were regularly monitored during the experiment. After the indicated days post-injection, the mice in euthanasia cages were euthanized by filling the cage with CO2 at a rate of 30–70% of container volume per minute at an equilibrium rate. Once the mice were unconscious, we also performed cervical dislocation to minimize their fear, anxiety, and pain. Buccal tumors and cervical lymph nodes were then harvested for weighing and immunohistochemical (IHC) staining. The antibody for Ki-67 was used as a cell proliferation marker, whereas that against pan-cytokeratin (Pan-CK) was used as an epithelial marker (Table S2).

#### Trans-endothelial migration

The transendothelial migration assay was carried out as described [27] with some modifications. HMEC-1 cells (150,000 cells/well) in triplicate on gelatin-coated Millicell culture inserts (8 μm pore size) reached confluence 3 days after seeding. We seeded vector or UCA1-manipulated HPC cells (10,000 cells per well) onto the inserts with TNF-α-activated or untreated endothelial monolayer (a negative control). They were allowed to migrate for 24 h to the bottom wells. The tumor cells that migrated to the lower surface were stained with 0.2% crystal violet in 20% methanol and enumerated in high-power fields under a microscope.

#### RNA immunoprecipitation (RIP) assay

We performed RIP assay as described [28]. Briefly, FaDu cells were lysed in a buffer containing 20 mM MgCl_2_, 1.28 M sucrose, 4% Triton X-100 and 40 mM Tris-HCl (pH 7.5). We then incubated lysates on ice for 20 min followed by centrifugation for 15 min at 2,500 x g to pellet nuclear lysates. The nuclear pellet was homogenized in the RIP buffer containing 25 mM Tris-HCl (pH 7.4), 150 mM KCl, 5 mM EDTA, 0.5% NP-40, 0.5 mM DTT, protease inhibitors and RNAase inhibitor (100 U/ml), then centrifuged for 10 min at 16,200 x g to collect the supernatant. The nuclear supernatant was incubated with an anti-PTBP3 antibody (Table S2), or control IgG overnight at 4 ° C with gentle rotation. The immunocomplex was collected with protein G agarose beads, followed by three washes with RIP buffer. RNA was extracted from the immunocomplex with TRIzol reagents and assayed by RT-qPCR.

#### Hypoxic treatment

The indicated cells were exposed for the indicated Hours to 1% O_2_ in the hypoxic chamber prior to the harvest of total RNA and proteins for RT-qPCR, Western blots, or luciferase reporter assays.

#### Statistical analysis

The numerical data were expressed as mean ± SD of 3 independent experiments of cell-based assays, whereas mean ± SEM was used for in vivo experiments. Following performing paired Student’s t-test or One One-Sample T-test for the normalized data, statistical significance was defined as * *p*<0.05, ** *p*<0.01, *** *p*<0.001.

#### Online supporting methods

Other methods can be found in Supplementary Information 1.

## Results

### Differential expression of UCA1 lncRNA in HPC tissues and head and neck cancer cell lines

The *UCA1* gene encodes four experimentally validated isoforms [14, 15, 29]. Additionally, one isoform has been identified and assembled by the NCBI (NR_015379.3) (Fig. S1A). Analyses of Deepbase v3.0 [30] reveal an increased mean *UCA1* expression across various cancer types, including head and neck squamous carcinoma (HNSC) (Fig. S1B). We investigated further by comparing *UCA1* expression from the pairwise normal and cancerous tissues of three HPC patients. Our results showed a significant increase in *UCA1* expression in tumors compared to normal counterparts (Fig. S1C). We also found a significant increase of UCA1 in most HNC lines when compared to human normal oral keratinocytes (HNOK) (Fig. S1D). Due to limited HPC lines, we chose HPC-derived FaDu and pharynx-derived Detroit 562 cells for overexpression and knockdown studies, respectively.

### UCA1 knockdown attenuates cell migration and invasion without a significant effect on cell proliferation

**Fig. 1:**
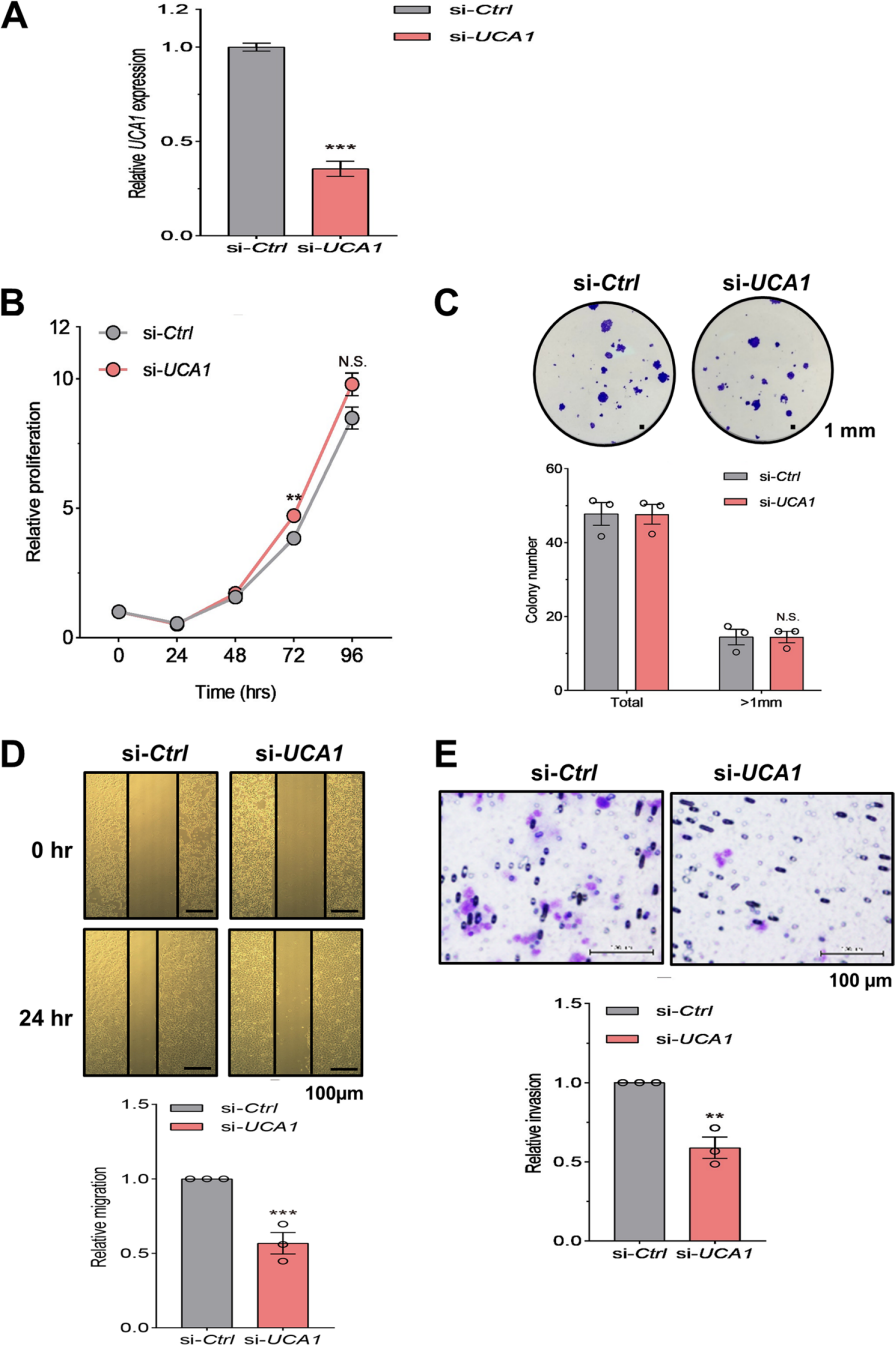
*UCA1* depletion inhibits HNC cell migration and invasion with a Marginal effect on their proliferation. Detroit 562 cells were transfected with si-*UCA1* or si-Control (si-Ctrl). Following validation of the reduced *UCA1* expression by RT-qPCR (**A**), we assayed cell proliferation (**B**), colony formation (**C**), migration (**D**) and invasion (**E**). All the experiments were independently repeated three times and expressed as mean ± SD (*N* = 3). ** *p*<0.01 or not significant (N.S.) compared to si-Ctrl, t-test. N.S., not significant

To determine the role of *UCA1* in pharyngeal cancer cells, we carried out knockdown experiments in Detroit 562 cells, which express high levels of endogenous *UCA1*. The knockdown efficiency reached nearly 70% of *UCA1* depletion, as shown by RT-qPCR analysis (Fig. 1A). Interestingly, despite lacking a significant effect on the proliferation and colony formation (Fig. 1B-C), *UCA1* knockdown significantly reduced cell migration (Fig. 1D) by using wound healing assay, and invasion (Fig. 1E) by using Transwell invasion assays (See Supplementary Information 1 for details). The effect of UCA1 depletion on cell proliferation, migration and invasion was also validated by using CRISPR interference-mediated suppression of *UCA1* expression (see Supplementary Information 1 for details and Fig. S2). Together, these results revealed that *UCA1* can promote migration and invasion in pharyngeal cancer cells.

### Ectopic UCA1 expression promotes cell migration and invasion while inhibiting cell proliferation

**Fig. 2:**
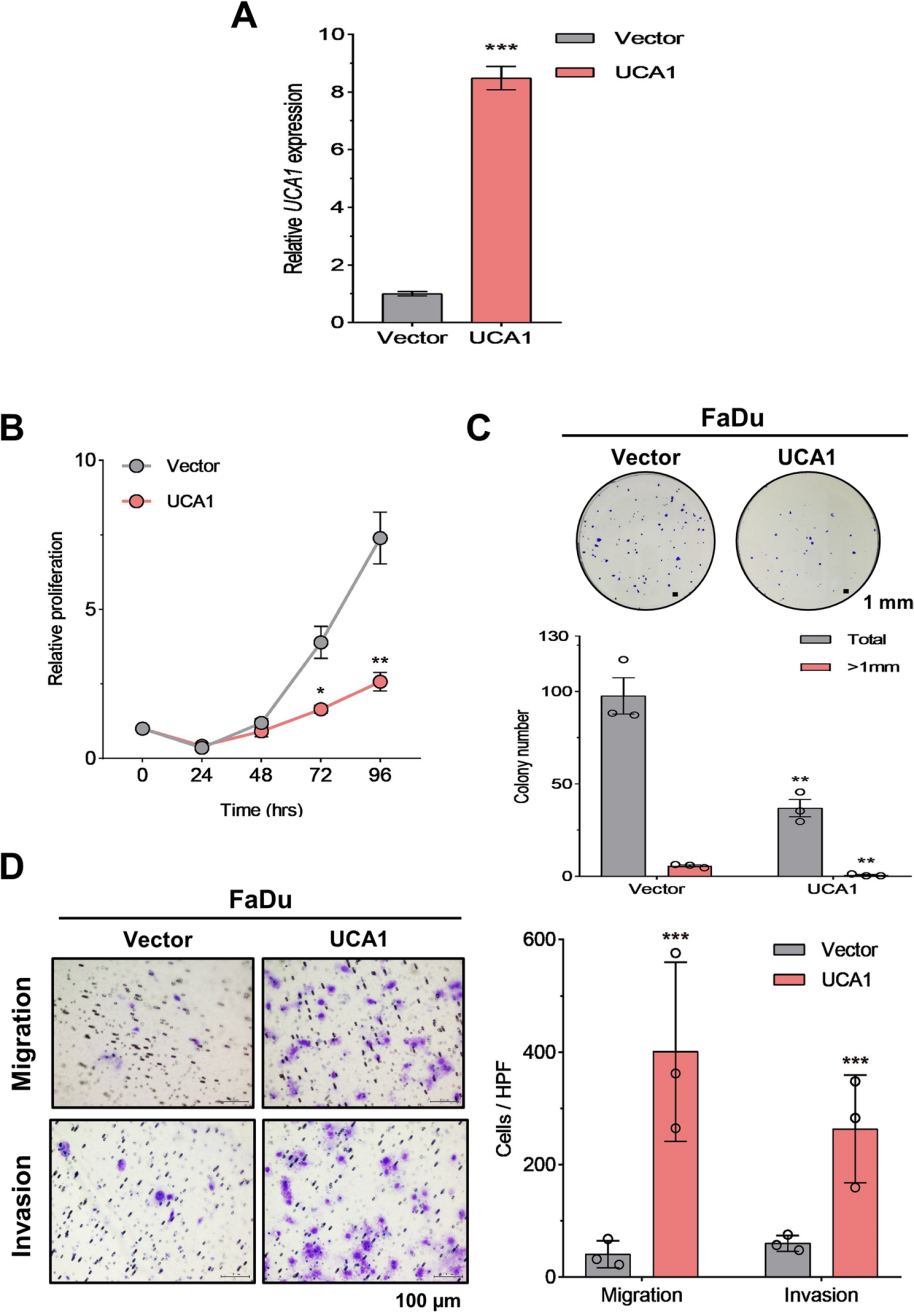
*UCA1* overexpression promotes cell migration and invasion while reducing cell proliferation. FaDu cells were transduced with pLKO-UCA1-1.4 kb or vector-bearing lentivirus. Following validation of the increased *UCA1* expression (**A**), we assessed cell proliferation (**B**), colony formation (**C**), as well as migration (**D**) and invasion (**E**) using cell culture inserts without and with Matrigel coating, respectively. All the experiments were independently repeated three times and expressed as mean ± SD (*N* = 3). * *p* < 0.05, ** *p* < 0.01, *** *p* < 0.001 or N.S. compared to vector, t-test

The 1.4-kb *UCA1* isoform is sequence-validated [14, 29] and is oncogenic in bladder cancer [29] (Fig. S1A). We further performed isoform-specific qPCR and showed that the 1.4-kb *UCA1* is the predominant form in various head and neck cancer cell lines relative to those in HNOK (Fig. S3A). We cloned the 1.4-kb *UCA1* and established FaDu cell lines stably overexpressing *UCA*1. The levels of overexpressed *UCA1* were confirmed by RT-qPCR (Fig. 2A). Next, we assessed the effects of UCA1 overexpression on proliferation, colony formation, as well as migration and invasion using the Transwell migration and invasion assay (See Supplementary Information 1 for details). We observed an increase in migration and invasion while suppressing proliferation and colony formation in *UCA1*-expressing FaDu cells (Fig. 2B-E). These data are partly consistent with the knockdown results and indicate a promoting role of the 1.4-kb *UCA1* in cell migration and invasion.

### Overexpression of UCA1, enhancing the expression of several EMT markers, mimics the effect of TGF-β on gene expression and promotes trans-endothelial migration

**Fig. 3:**
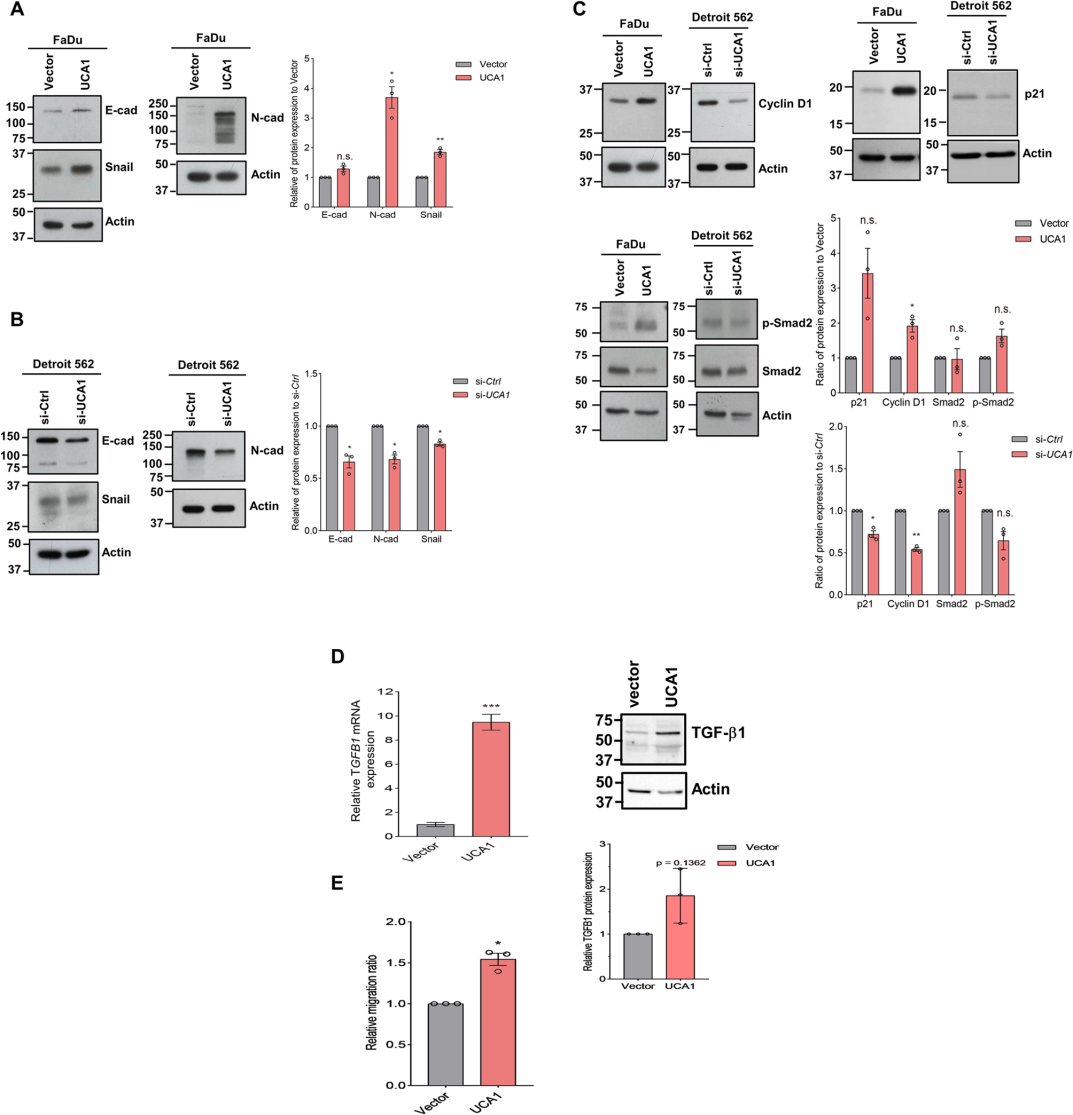
A concordant change in the expression of EMT markers with *UCA1* dysregulation which mimics the effect of TGF-β and regulates trans-endothelial migration. Epithelial marker (E-cad) and mesenchymal markers (N-cad and snail) were measured by Western blot analysis in the *UCA1* (**A**) or si-*UCA1* expressing cells (**B**) as well as control cells. Ratio of the indicated protein expression relative to control are shown in bar charts (*N* = 3). (**C**) Top, Western blot analysis of cyclin D1 and p21 in the *UCA1*-manipulated FaDu or Detroit 562 lines. Bottom, the activating phosphorylation at Ser465/467 of Smad2 (p-Smad2) and its total level in UCA1-manipulated cells. Actin serves as a loading control. The ratio of the indicated protein expression relative to the control are shown in bar charts (*N* = 3). (**D**) The relative expression of *TGFB1* mRNA (Left) and protein (Right) in FaDu cells expressing vector or UCA1, respectively measured by RT-qPCR and Western blot analysis, shown in bar charts (*N* = 3). *** *p*<0.001 compared to Vector. (**E**) The relative ratio of cells migrating across endothelial monolayers. The experiments were independently repeated three times and expressed as mean ± SD (*N* = 3). * *p*<0.05, or ** *p*<0.01 compared to vector or si-Ctrl. N.S., not significant. The blot images are presented in Supplementary Figures S7-S13

Our cell migration and invasion assays suggest a potential role of *UCA1* in promoting epithelial-mesenchymal transition (EMT), a process related to cancer metastasis [31]. To dissect the underlying mechanism by which *UCA1* promotes EMT, we examined if manipulating *UCA1* abundance affected the expression of EMT markers. Our data showed that N-cadherin (N-cad) abundance was significantly increased, accompanied by a marginal increase in the expressions of E-cadherin (E-cad) and Snail in *UCA1*-overexpressing FaDu cells (Fig. 3A). The opposite results on the expression of EMT markers were observed in *UCA1* knockdown Detroit 562 cells (Fig. 3B). Collectively, these results imply that increased *UCA1* expression induces incomplete EMT [32] through increasing the abundance of mesenchymal marker proteins without reducing the expression of epithelial markers.

Like *UCA1*, TGF-β, a master regulator of the EMT process, suppresses tumor growth but promotes metastasis [33]. TGF-β induces the expression of cyclin D1 and p21 while suppressing oral cancer cell proliferation [34]. We therefore examined whether altered *UCA1 *expression would affect cyclin D1 and p21 expression, similar to TGFβ stimulation. Indeed, ectopic *UCA1*-expressing cells showed increased cyclin D1 and p21 protein levels, while *UCA1 *silencing had the opposite effects (Fig. 3C). Smad2 phosphorylation at Ser465/467 is one downstream module of the canonical TGF-β signaling pathway [33]. We also observed a significant increase in Smad2 phosphorylation (Fig. 3C) as well as in the expression of TGFB1 mRNA and protein (Fig. 3D) in UCA1-overexpressing cells, further supporting the role of *UCA1 *in mimicking TGF-β in head and neck cancer cells.

Trans-endothelial migration of tumor cells is a complex process that involves adhesive interactions between endothelium and tumor cells [35]. N-cadherin was previously shown to play a role in trans-endothelial migration of melanoma cells [27], and *UCA1* manipulation affected the expression of N-cadherin (Fig. 3A). To separate the negative effect of *UCA1* expression on cell proliferation from cell migration and invasion, we used trans-endothelial migration assay, a co-culture scheme mimicking the process of tumor cell extravasation, to examine the effect of *UCA1* overexpression on this process. As shown in Fig. 3E, increased *UCA1* expression induces a concordant increase in head and neck cancer cell migration through endothelial monolayer.

### UCA1 silencing reduces metastasis while overexpression inhibits tumor growth

**Fig. 4:**
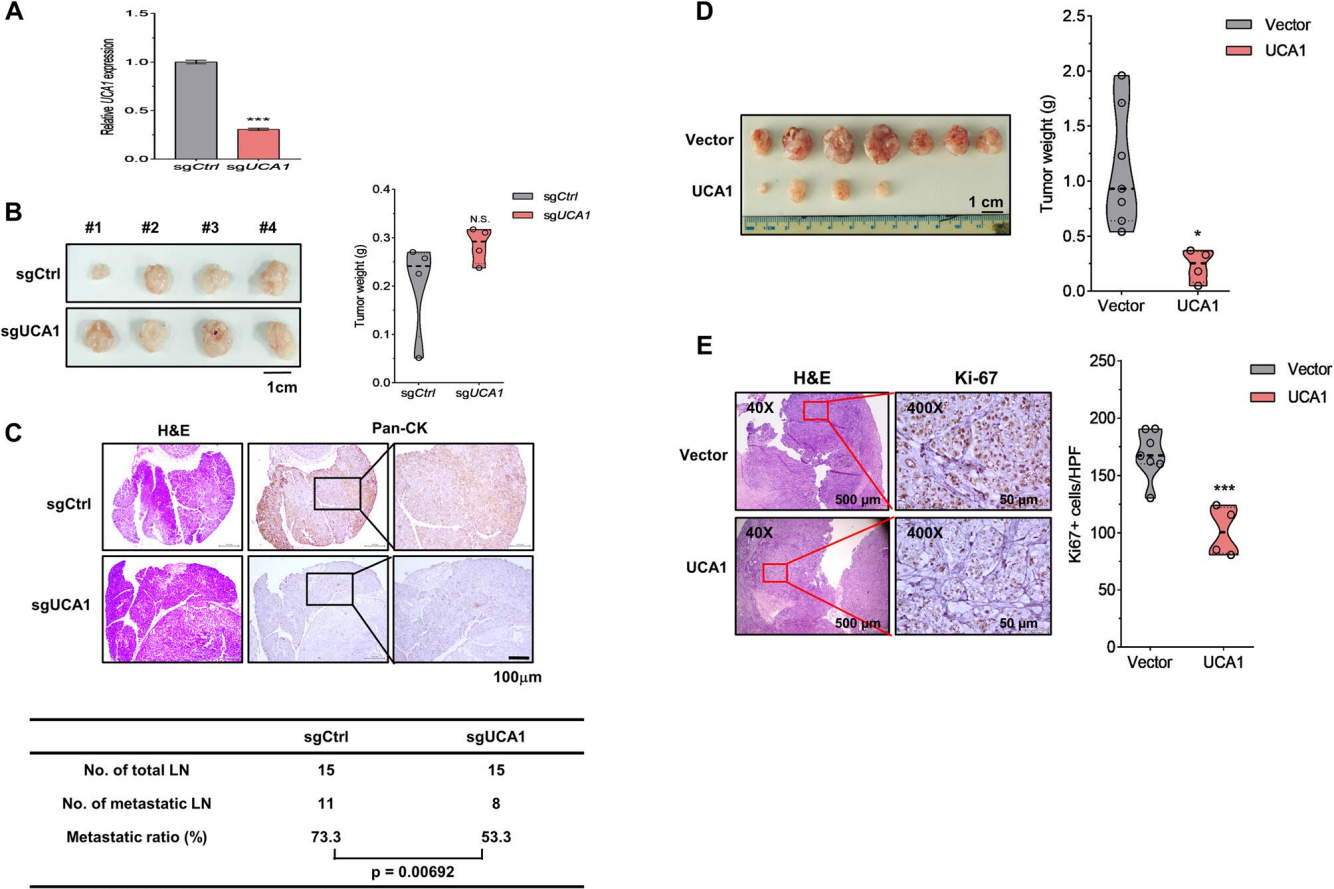
*UCA1* depletion reduces lymph node metastasis in vivo. (**A**) RT-qPCR analysis of *UCA1* expression in sgCtrl and sgUCA1-bearing Detroit 562 cells. (**B**) The image of primary tumors (sgCtrl versus sgUCA1) and their weights at the buccal sites 34 days after injection (*N* = 4 per group). (**C**) Top, H&E and IHC staining of pan-CK positivity in the cervical lymph nodes harvested from sgCtrl and sg*UCA1* tumor-bearing mice. Bottom, the number of metastatic lymph nodes (LN) was expressed as the percentage of total LN in each mouse group. (**D**) The image of primary FaDu tumors bearing empty vector or *UCA1* in the buccal sites and their weights at 44 days after injection (*N* = 7 per group). (**E**) H&E and IHC staining of Ki67 positivity in tumor sections, respectively, harvested from vector and *UCA1* tumor-bearing mice. * *p* < 0.05 or *** *p* < 0.001 versus vector, t-test. N.S., not significant

To evaluate the in vivo role of *UCA1*, we used a xenograft mouse model via buccal injection of stable Detroit 562 transfectants expressing sgCtrl (control) or sg*UCA1* (*UCA1*-depletion) into male NOD-SCID mice following the validation of successful depletion of endogenous *UCA1* by RT-qPCR (Fig. 4A). *UCA1* depletion modestly increased primary tumor weights (Fig. 4B). We also collected the cervical lymph nodes around buccal tumors to examine the presence of pan-cytokeratin (pan-CK)-positive tumor cells in the nodes by using IHC staining. *UCA1* silencing significantly diminished the lymphatic invasion of tumor cells stained by anti-pan-CK antibody (Fig. 4C), suggesting that *UCA1* promotes metastasis in vivo. Consistent with our in vitro findings and a positive impact of tumor cell proliferative capacity on their metastasis propensity [36, 37], *UCA1* overexpression in Detroit 562 cells could potently suppress tumorigenesis as well as Ki67 + tumor cell proliferation in mice (Fig. 4D-E) and their seeding abilities in lymph nodes (data not shown).

### PTBP3 associates with UCA1 in the nucleus

**Fig. 5:**
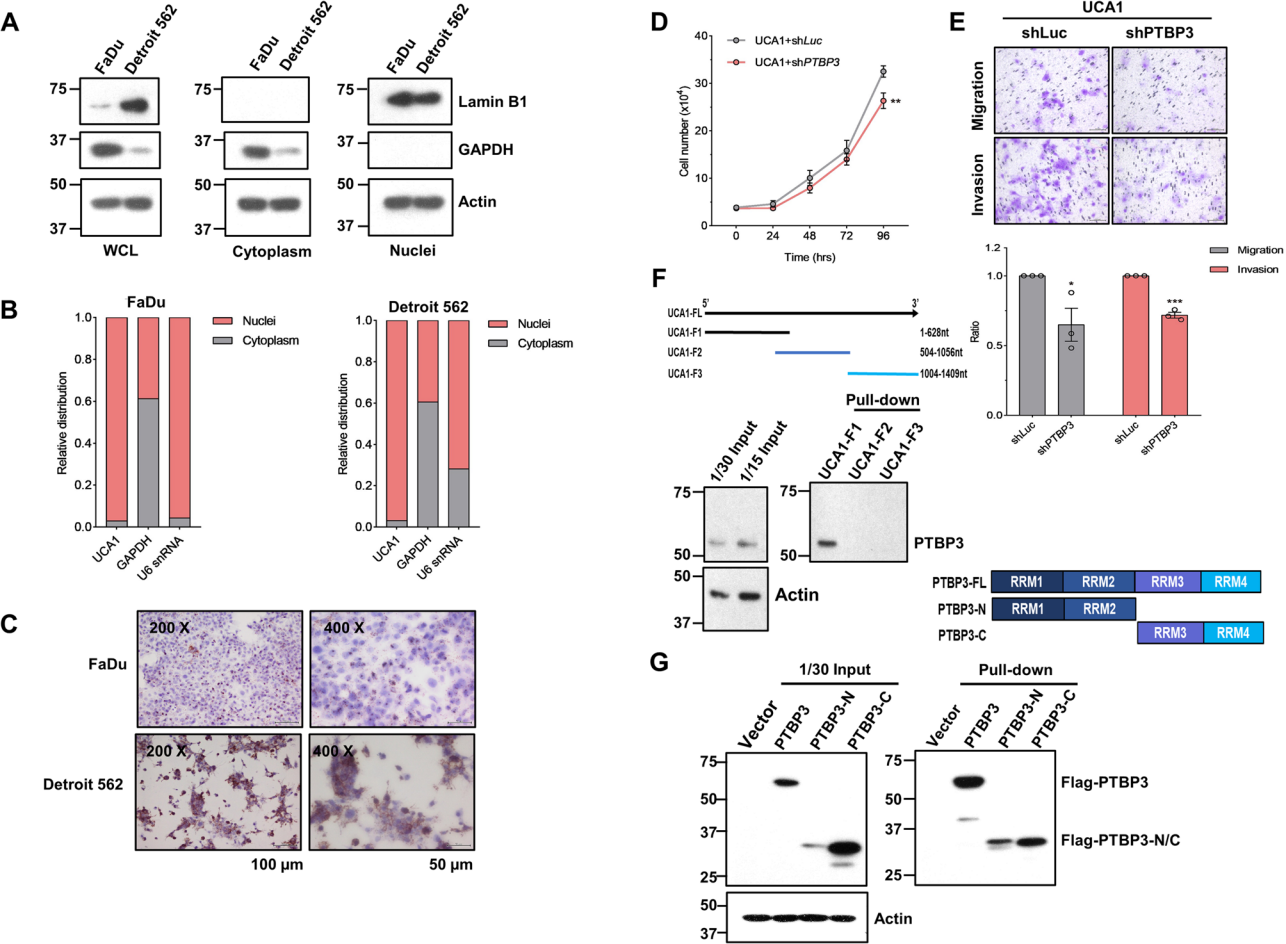
*UCA1*, primarily localized in the HNC cell nuclei, interacts with PTBP3 that modulates *UCA1*-mediated cell proliferation, migration and invasion. (**A**) Western blot analysis of nuclear (lamin B1) and cytoplasmic (GAPDH) in the whole cell lysates (WCL) and subcellular fractions. Actin as a loading control. This result is a representative of three independent repeats. (**B)** The levels of *UCA1* in the indicated fractions from FaDu and Detroit 562 lines by RT-qPCR. *GAPDH* and *U6* snRNA are, respectively, used as a cytosolic control and a nuclear control. (**C)** *UCA1* expression in FaDu and Detroit 562 lines was detected by ISH. Brown, *UCA1* staining positivity. Blue, nuclear counterstain by haematoxylin. The effect of *PTBP3* silencing on cell proliferation (**D**) and migration and invasion (**E**) of *UCA1* ectopically expressed cells. ** *p* < 0.01 or *** *p* < 0.001 compared to shLuc cells, t-test. (**F**) The 1.4 kb *UCA1* is divided into three segments for RNA pulldown assays (Top). Bottom, PTBP3 preferentially binds to the first 628 nt of *UCA1*−1.4 kb (UCA1-F1). Actin is the loading control. (**G)** PTBP3 protein is divided into PTBP3-N and PTBP3-C as indicated (Top). *UCA1* binds to both PTBP3-N and PTBP3-C (Bottom). This result is a representative of two independent repeats. The full-length blots are presented in Supplementary Figures S14-S16

The subcellular distribution of lncRNAs is vital to their biological functions [20]. To delineate the mechanism by which nuclear *UCA1* regulates cellular function, we examined the subcellular localization of *UCA1* in FaDu and Detroit 562 cells by subcellular fractionation. We also performed Western blot analysis of whole cell, cytoplasmic, and nuclear lysates to validate nucleocytoplasmic fractionation (Fig. 5A). RT-qPCR analyses revealed a predominantly nuclear localization of *UCA1* (Fig. 5B). Furthermore, ISH showed the presence of *UCA1* in the blue nuclei stained by haematoxylin, a nuclear counterstain, in both FaDu and Detroit 562 cell lines (Fig. 5C). These results showed that *UCA1* is predominantly localized in the nuclei of pharyngeal cancer cells.

Next, we performed RNA Pulldown using biotinylated 1.4-kb *UCA1* as bait to identify its associated proteins. Nuclear lysates prepared from FaDu cells were incubated the lysates with biotinylated-*UCA1* or its antisense RNA (negative control) (Fig. S4A), followed by mass spectrometry, and PTBP3 was identified as one of the *UCA1*-interacting partners. The interaction between PTBP3 and *UCA1* was confirmed, respectively, by RNA pulldown and RNA immunoprecipitation (RIP), followed by Western blotting (Fig. S4B-C). To examine the role of PTBP3 in UCA1-mediated cellular activity, we silenced the expression of PTBP3 in *UCA1*-overexpressing FaDu cells and assayed its effects on cell proliferation, migration, and invasion. Our results showed that PTBP3 silencing attenuated cell proliferation, migration, and invasion in the presence of ectopic *UCA1* expression (Fig. 5D-E). By contrast, ectopic PTBP3 overexpression could rescue *UCA1*-mediated suppression of cell migration and invasion (Fig. S5). Together, these findings suggest a positive role of PTBP3 in *UCA1*-mediated migration and invasion. The reciprocal interacting domain was Mapped in the first 504bp of UCA1-1.4 kb and the entire PTBP3 protein by using RNA-pulldown and RIP assays (Fig. 5F-G).

### Hypoxia induces UCA1 expression through the distal hypoxia-responsive element (HRE) in UCA1 proximal promoter

**Fig. 6:**
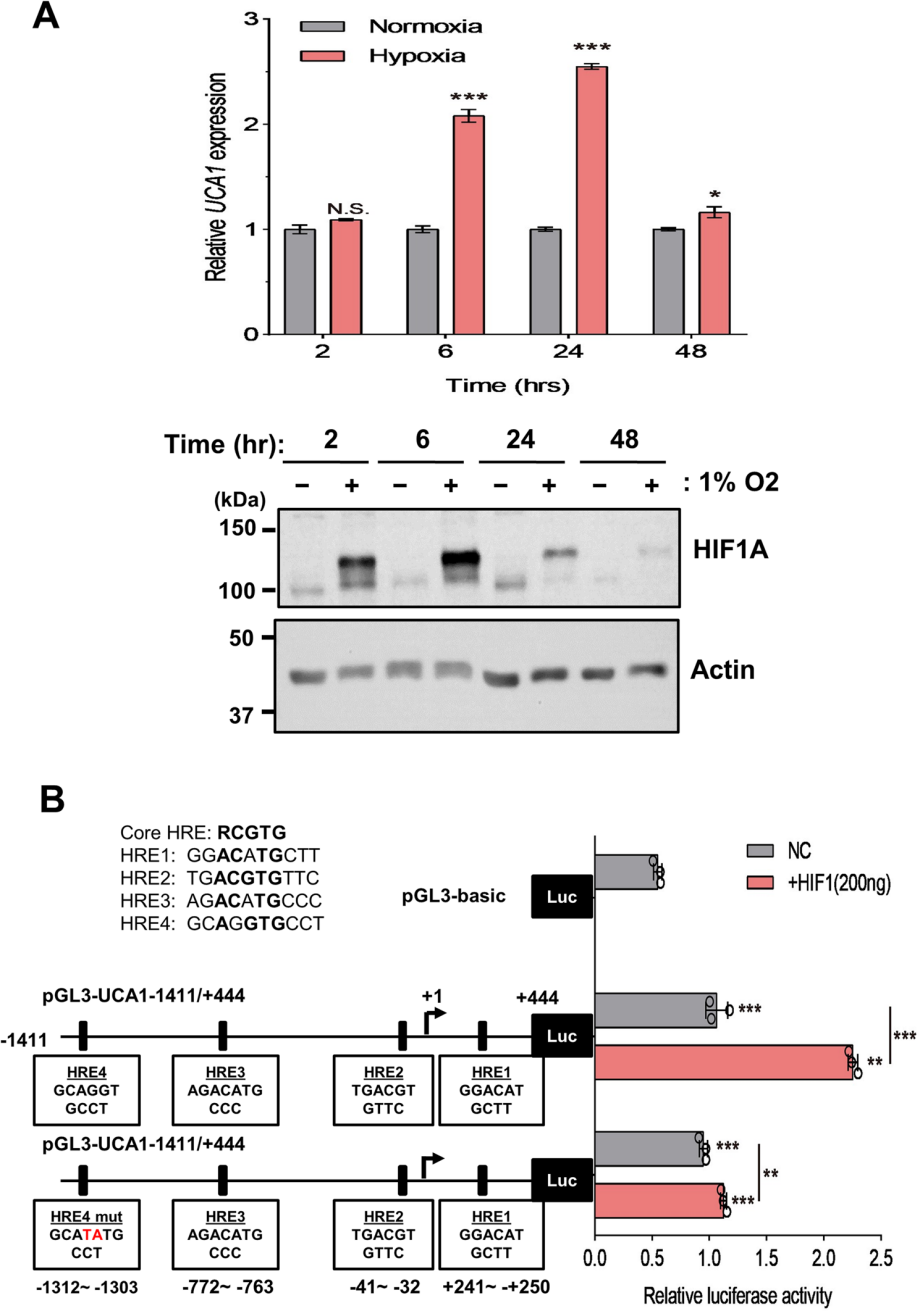
Regulation of *UCA1* expression by HIF1A. (**A**) Hypoxia induces HIF1A protein abundance and *UCA1* expression. Relative *UCA1 *expression by RT-qPCR (top) and HIF1A expression by Western blot analysis (bottom) in the FaDu cells under normoxia or hypoxia (1% O_2_) for 2-48 hrs. Actin serves as a loading control. This result is representative of three independent repeats. (**B**) Mutations in HRE4 markedly reduced HIF1A-induced *UCA1* promoter activity in a transient transfection reporter assay. +1 as transcription start site. ** p< 0.01 or *** p ＜ 0.001 compared to pGL3-basic vector, t-test. The experiments were independently repeated three times. Full-length blots are presented in Supplementary Figure S17

HIF1A is a major transcription factor that regulates transcription to promote tumorigenesis and metastasis in hypoxia, a cancer hallmark [38]. To test this possibility, we examined whether hypoxia induces *UCA1* expression by treating FaDu cells with a hypoxic condition at 1% O2 followed by RT-qPCR analyses. Indeed, hypoxia induces *UCA1* expression (Fig. 6A, top panel) following the elevation of HIF1A protein levels (Fig. 6A, bottom panel). Analyses of the proximal *UCA1* promoter sequence (−1411/+444) suggest that it harbors several putative hypoxia response elements (HRE1-4) (Fig.6B, left). Our reporter assays indicated that HIF1A increased the reporter activity in a dose-dependent manner (Fig. S6). Notably, a mutation in the distal HRE4 (HRE mut) significantly attenuated HIF1A-mediated induction of *UCA1* promoter activity (Fig. 6B, right), suggesting that HRE4 is subject to HIF1A regulation.

## Discussion

**Fig. 7:**
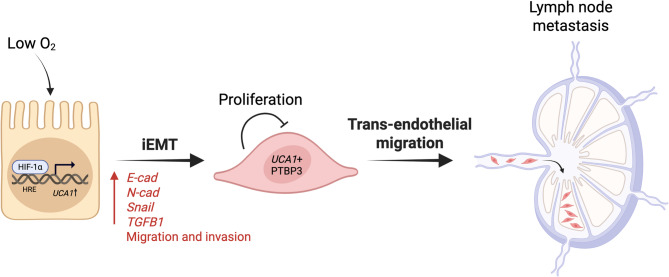
A model illustrating how hypoxic conditions induce *UCA1* expression through HIF-1a binding to HREs within its promoter. Consequently, *UCA1* up-regulation promotes multiple hallmarks of cancer progression, including incomplete epithelial-mesenchymal transition (iEMT) through activation of key regulatory genes, thereby enhancing cell proliferation, migration, invasion, and lymph node metastasis. HRE, hypoxia response element. Created with BioRender.com

Although *UCA1* expression in head and neck cancer is linked with advanced clinical stage, lymphatic invasion, and poor prognosis [22], the underlying mechanism involved in *UCA1*-mediated tumorigenesis remains unclear. In this study, we found that UCA1 levels were higher in HPC tissues than in adjacent normal tissues. Interestingly, while dampening cell proliferation, ectopic expression of the 1.4-kb *UCA1* could promote cell migration and invasion, and *UCA1* depletion had the opposite outcome. Furthermore, animal xenograft studies indicated a suppressive role of *UCA1* in primary tumor growth, whereas *UCA1* overexpression increased trans-endothelial migration, a process mimicking extravasation. These results suggest that *UCA1*’s role in cancer mirrors some activities of TGF-β. We further identified an association of *UCA1* with PTBP3 in the nucleus. Importantly, the knockdown of *PTBP3* resulted in significantly reduced migration and invasion, while overexpression rescued the cell migration and invasion of *UCA1*-knockdown cells. Lastly, we showed that hypoxia induces *UCA1* expression in HPC cells, possibly through the HREs in its proximal promoter (Fig. 7).

Although *UCA1* has been implicated to be oncogenic in certain cancer types [13, 15–19], our data indicate an inhibitory effect of *UCA1* on proliferation, colony formation, and tumor growth in HPC while promoting migration and invasion (Figs. 2 and 3). In support of the role of *UCA1* in promoting migration and invasion, we observed a positive correlation between *UCA1* expression and 4-gene EMT signature (Fig. S3B, *r* = 0.12, *p* < 0.01) in TCGA-HNC tumor tissues by GEPIA2 analysis. This correlation was further validated by Western blot analysis (Fig. 3). Similar dual effects of *UCA1* on cell proliferation and migration/invasion have been reported in oral and breast cancer cells treated with TGF-β, a master regulator of EMT [33, 34]. TGF-β suppresses tumor formation in early stages but promotes metastasis [39]. Takahashi et al. recently showed the appearance of a motile population of mesenchymal oral cancer cells upon TGF-β-induced cell-cycle arrest in G1 [34]. Notably, we also detected that manipulation of *UCA1* expression had a similar effect to TGF-β on the expression of both cyclin D1 and p21, as well as activated Smad2 (p-Smad2), possibly via inducing TGFB1 expression (Fig. 3D), indicative of the promoting effect of *UCA1* expression on motile phenotype. Differences in the extent of TGFB1 expression at both the mRNA and protein levels are supported by numerous studies, which demonstrate that TGF-β is subject to complex regulation at multiple stages, including the translational and post-translational levels [40].

Since the functions of lncRNAs are impacted by their subcellular locations [20], the discrepancy between our findings and those in oral squamous cell carcinoma [13] can be attributed to a distinct subcellular location, nuclei versus cytosol, respectively, in our and their studies. Moreover, both oral cell lines used in their studies are later shown to be HeLa derivatives, cervical adenocarcinoma [41].

EMT is a crucial step required for metastatic spread [42]. The ability of metastatic cells to gain an incomplete, rather than a complete, EMT phenotype poses a higher risk of metastasis in cancer patients [32, 43]. Incomplete EMT is primarily characterized by the simultaneous presence of both epithelial and mesenchymal markers. Cancer cells can switch between proliferation and motility, two contrasting events during tumor progression [44, 45], in response to challenging and dynamic microenvironments, such as hypoxia, acidity, or limited nutrient availability [46]. Under such conditions, proliferating tumor cells tend to be less invasive, whereas invasive cells are less proliferative [47, 48]. In line with these observations, UCA1 manipulation had the opposite effect in vitro and in vivo on cell proliferation, migration, and invasion (Figs. 1 and 2, and 4). To overcome the complication from a negative effect on cell proliferation, we adopted a trans-endothelial migration assay, which mimics extravasation, to examine the effect of *UCA1* expression. Consistent with the in vitro migration and invasion assays, *UCA1* expression could increase the extravasating ability of head and neck cancer cells (Fig. 3D), consistently supporting a promoting role of *UCA1* overexpression in metastatic spread.

Increasing evidence showed that lncRNAs differentially regulate gene expression depending on their cellular localizations [20]. Among them, nuclear lncRNAs participated in the epigenetic and transcriptional regulation of neighboring or distal genes [49]. Cytosolic lncRNAs regulate mRNA stability and translation, serve as competing endogenous RNAs for sponging miRNAs, and mediate protein modifications [50]. In addition, their interactions with RNAs, DNAs, or protein complexes can affect several cellular functions of great physiological relevance. The alteration of their expressions is thus inherent to many diseases [20]. *UCA1* was previously reported in the cytoplasm of bladder cancer and glioma cells [16, 17]. In contrast to these studies, we found a predominant nuclear localization of *UCA1* in pharyngeal cancer cells through the analysis of subcellular fractionation followed by RT-qPCR and ISH **(**Fig. 5A-C**)**.

LncRNAs in blood circulation have shown great promise as cancer biomarkers [10]. The intracellular distribution of lncRNAs affects their diverse function; however, it remains elusive whether the release of nuclear lncRNAs into the bloodstream is a regulated and specific process for certain lncRNAs or a more generalized phenomenon, especially in disease states. Although we did not conduct a systematic analysis of showing nuclear UCA1 of being a biomarker in this report, it has been shown that MALAT1, also a nuclear lncRNA, could be secreted to impact immune cells in the same microenvironment via exosomes [51]. In support of this observation, we also detected the accompanying increase of *UCA1* in the conditioned medium derived from *UCA1*-overexpressing cells relative to vector control (data not shown), suggesting the possibility of using an lncRNA like *UCA1* as a biomarker.

We have identified PTBP3 as a *UCA1*-interacting partner. Loss of *PTBP3* by knockdown could further reduce migration and invasion in *UCA1*-overexpressing cells, while ectopic PTBP3 expression partially rescued *UCA1* knockdown-mediated reduction in migration and invasion **(**Figs. 5 and S5), in support of their involvement in the EMT process. Indeed, PTBP3 is crucial for TGF-β-mediated EMT and metastatic spread of lung cancer cells [52]. Interestingly, PTBP3 binds to the internal ribosome entry site of *HIF1A* mRNA to regulate HIF1A translation, thereby promoting colorectal cancer cell proliferation [53]. Cooperative crosstalk between TGF-β and hypoxia has been reported in lung and prostate cancer [54]. TGF-β can thus activate HIF targets in normoxic conditions and promote their expression in response to hypoxia. This synergistic loop was shown to facilitate the development of aggressive cancer in the prostate [55]. In the same line of observations, we found that HIF1A-induced PTBP3-bound *UCA1* expression, mimicking the effect of TGF-β, mediated cell migration and invasion in head and neck cancer cells. These observations warrant an in-depth investigation of the interplay between TGF-β-signalling and *UCA1*.

## Conclusions

In summary, *UCA1* lncRNA inhibits head and neck cancer cell proliferation; however, it can promote migration and invasion of head and neck cells, by inducing incomplete EMT, including upregulating the expressions of E-cad, N-cad, SNAIL and TGFB1. Hypoxia, together with the interaction with the PTBP3 protein, can contribute to the increase in *UCA1* expression in head and neck cancer cells (Fig. 7). More molecular studies will be warranted to determine whether *UCA1* can be used as a therapeutic target against HPC.

## Supplementary Information

Supplementary Material 1.

Supplementary Material 2. Tab. S1. Primers and probes

Supplementary Material 3. Tab. S2. Antibody list

Supplementary Material 4. Fig. S1. Differential expression of *UCA1* in the indicated cancer tissues or cell lines

Supplementary Material 5. Fig. S2. CRISPR/Cas-mediated silencing of *UCA1* expression recapitulated the depletion effect of *UCA1* by using siRNA on cell proliferation, migration, and invasion.

Supplementary Material 6. Fig. S3. The expression of*UCA1* isoforms in the HNC lines and a positive correlation of *UCA1* with mesenchymal signatures in the TCGA-HNC database

Supplementary Material 7. Fig. S4. PTBP3 is identified as a *UCA1*-interacting partner

Supplementary Material 8. Fig. S5. Ectopic PTBP3 overexpression rescues *UCA1*-mediated suppression of cell migration and invasion

Supplementary Material 9. Fig. S6. HIF1A potentiates *UCA1* promoter activity in a dose-dependent manner.

Supplementary Material 10.

## Data Availability

All data generated or analysed during this study are included in this published article and its supplementary information files. Further inquiry can be directed to the corresponding author.

## Abbreviations

HNC: Head And Neck Cancer
HPC: Hypopharyngeal Cancer
lncRNA: Long Noncoding RNA
UCA1: Urothelial Cancer Associated 1
EMT: Epithelial Mesenchymal Transition
HIF1A: Hypoxia Inducible Factor 1A
PTBP3: Polypyrimidine Track Binding Protein 3
