# Supplementary material for: Targeting the HIF1A-UCA1-PTBP3 axis: a potential therapeutic strategy for head and neck cancer
Source: BMC Cancer. 2025 Oct 9;25:1536. doi: 10.1186/s12885-025-15020-z (PMC12512865; doi:10.1186/s12885-025-15020-z)
Supplement: Supplementary file 1 — Supplementary Material 1. [file 12885_2025_15020_MOESM1_ESM.pdf]

Targeting the HIF1A-UCA1-PTBP3 axis: A potential therapeutic strategy for head and neck cancer

Lydia Chin-Ling Sim[1†], Yi-Zih Kuo[1,2†], Tsun-Chih Cheng[3†], Chih-Ling Chen[1], Jenn-Ren Hsiao[2], Mei-Xuan Ooi[1], Zixi Yun[4], Hung-Ying Kao[4], Sen-Tien Tsai[2,5*], and Li-Wha Wu[1,6*]

[1] Institute of Molecular Medicine, College of Medicine, National Cheng Kung University, Tainan, Taiwan, R.O.C.

[2] Department of Otolaryngology, National Cheng Kung University Hospital, College of Medicine, National Cheng Kung University, Tainan, Taiwan, R.O.C.

[3] Department of Otolaryngology, An Nan Hospital, China Medical University, Tainan, Taiwan, R.O.C.

[4] Department of Biochemistry, School of Medicine, Case Western Reserve University, Cleveland, OH, U.S.A.

[5] Research Department, Changhua Christian Hospital, Changhua City 50006, Taiwan, R.O.C.

[6] Department of Laboratory Science and Technology, College of Health Sciences, Kaohsiung Medical University, Kaohsiung, Taiwan, R.O.C.

†Equal contribution

*Correspondence:

Li-Wha Wu

ORCID: 0000-0001-7010-6220

liwhawu@mail.ncku.edu.tw

Sen-Tien Tsai

T602511@mail.ncku.edu.tw

Supporting methods

RNA isolation and RT-qPCR

We harvested the total RNA from the indicated cells using the TRIzol reagent. One microgram of the isolated RNA was reversely transcribed to cDNA using a High-Capacity Reverse Transcription kit (Thermo Fisher Scientific, Waltham, MA, USA). The cDNA was used as the template for real-time PCR by using SYBR GREEN PCR Master Mix (Thermo Fisher Scientific, Waltham, MA, USA). *UCA1*, *GAPDH* (cytoplasmic control), or *U6* snRNA (subnuclear marker) were amplified, and the signals were quantified using the $2^{-\Delta Ct}$ method following the normalization by *GAPDH* or *U6* snRNA. We used the 2−ΔΔCt method to quantify the relative expression level of transcripts. The *UCA1* primer sequences targeting different *UCA1* isoforms are given in **Table S1**.

Patient specimens

The specimens of HPC, a rare form of head and neck cancer, were from 3 treatment-naive male patients undergoing surgery at NCKU Hospital. Written informed consent was provided by all participants. With the patients' informed consent, the biopsies of histologically proven normal tissue in the hypopharynx other than tumor sites were taken as pair-wise normal controls. All the fresh samples were handled anonymously, snap-frozen, and stored in liquid nitrogen until use. This study was conducted with the approval of the Institutional Review Board at National Cheng Kung University (A-ER-107-371). All participants gave consent to participate the study and for publication.

Plasmid construction for ectopic overexpression of UCA1 or PTBP variants

We individually constructed pLKO-based lentiviral vectors bearing UCA1-1.4 kb (Accession number: DQ343132.1), Flag-tagged PTBP3 or its variants (PTBP3-N or PTBP3-C) by PCR cloning, followed by sequencing validation. All these constructs were used for their ectopic overexpression in the cultured cells.

ShRNA-mediated PTBP3 silencing

PTBP-targeting shRNA (shPTBP3) and its control shLuc were purchased from RNAi Core at Academia Sinica (Taipei, Taiwan) for silencing PTBP3 gene expression in the cells.

Lentiviral preparation and transduction

The pLKO-based vector together with pCMV-Δ8.91 and pMDG-VSV-G were co-transfected for 48-72 hrs into human 293T cells with Lipofectamine 2000 (Thermo Fisher Scientific, Waltham, MA, USA), followed by the collection and titering of lentiviral particles in the culture media. To generate stable expressing clones, we infected cells with the indicated viruses at an MOI of 2 to establish the ectopic expression of UCA1 or PTBP3 in target cells.

CRISPR interference-mediated inhibition of *UCA1* expression

To sustain the depletion of *UCA1*, we also used CRISPR interference (CRISPRi) [1] to reduce endogenous *UCA1* expression in Detroit 562 cells and validated *UCA1* reduction in the manipulated cells by RT-qPCR. We suspended control (sgCtrl) or *UCA1*-depleted cells (sg*UCA1*) in phosphate-based saline and injected $1x10^5$ manipulated cells into murine buccal mucosa. Two guide RNAs were used for the cell-based assays. sg*UCA1* clone 2: GTGCATGGTGGAGAGATGAT; sg*UCA1* clone 3: TGGTTATCTGTTGTCAGCAG. Following validation of the *UCA1* silencing effect

on cell proliferation, migration, and invasions, only clone 2 was chosen for xenograft studies.

Western Blotting

We harvested the indicated cells in SDS lysis buffer (1% SDS and 10 mM Tris-HCl, pH 7.4). We measured the protein concentrations in the lysates using a Bio-Rad protein assay (Bio-Rad Laboratories, Hercules, CA, USA). We fractionated total proteins on SDS-PAGE, followed by electroblotting onto polyvinylidene fluoride membranes. The membranes were blocked for 1 hr in 5% non-fat milk, and then incubated overnight at 4°C with appropriate concentrations of antibodies and for 1 hr at room temperature with the HRP-conjugated secondary antibody. Protein bands were detected by a chemiluminescent immunodetection system and quantified by densitometry. β-actin served as a loading control. The detailed information on antibodies is shown in **Table S2**.

Cell proliferation assay

We measured the indicated cell proliferation by using a cell doubling assay. UCA1-manipulated and control cells seeded in 24-well plates were incubated at 37°C in a 5% CO2 incubator. Cells are detached by trypsin/EDTA for cell enumeration at 24, 48, 72, and 96 hrs following seeding. Alternatively, the proliferation of the indicated cells was assayed by using CellTiter 96® Aqueous One Solution Cell Proliferation Assay (Promega Corp., Madison, WI, USA).

Wound healing assay

The indicated cell lines were plated in 6-well plates pre-coated with 5 μg/ml collagen. After the seeded cells reached confluence, mitomycin C was added to block cell proliferation for 24 hrs. We then scratched the monolayer in the center with a 200 μl-

loading tip to generate the scratching wounds. The distance of the migrated cells was monitored through photography at the beginning and ending points under light microscopy. We measured the mean distance of ten wounds and calculated the migration rate (μm/hr).

Transwell migration and invasion assay

We performed this assay in 24-well Millicell culture inserts (Merck KGaA, Darmstadt, Germany) with 8-μm-pore polycarbonate membranes. The indicated cells ($3x10^5$/well) were seeded in triplicate into the upper chambers, in which the membranes were coated with or without 100 μg of Matrigel. We used the uncoated membranes for measuring cell migration ability for 16-24 hrs, but the coated ones for cell invasion for 48 hrs. Five hundred microliters of growth medium were added to the lower chambers. Upon the completion of either assay, the cells that migrated to the lower surface were stained with 0.2% crystal violet in 20% methanol and enumerated in high-power fields under a microscope.

Colony formation assay

We seeded and then incubated the indicated cells (200 cells per well) in 6-well plates at 37°C in a 5% CO2 incubator. Cell colonies appeared around 10-14 days and were washed several times with phosphate-based saline and stained with 0.2% crystal violet in 20% methanol. We counted the total colonies and those larger than 1 mm on the plates.

RNA pulldown and mass spectrometry analysis

In vitro transcription (IVT) of biotinylated RNA was generated by the MegaScript T7 Transcription kit with Biotin-11 UTP (Thermo Fisher Scientific, Waltham, MA, USA). Ten micrograms of biotinylated UCA1 or its antisense RNA were heated at 90°C and

chilled on ice, 2 min each, for proper RNA folding. The RNA structure buffer (0.1 M KCl, 10 mM MgCl2, and 10 mM Tris-HCl, pH 7.0) was added, followed by 20-min incubation at room temperature. To collect nuclear supernatant as described 26, we first lysed the indicated cells for 10 min at 4 °C with gentle rotation in a buffer containing 1.5 mM MgCl2, 10 mM KCl, 0.25% Nonidet P-40, 10 mM Tris-HCl (pH 7.0), 0.5 mM DTT, 1 mM PMSF, and a protease inhibitor cocktail (BIOTOOLS Co, New Taipei City, Taiwan). Following centrifugation, we resuspended the nuclear pellets with a buffer containing 0.5% Nonidet P-40, 150 mM KCl, 25 mM Tris-HCl (pH 7.0), 0.5 mM DTT, and a protease inhibitor cocktail. We sheared the pellets in a homogenizer with 25–30 strokes and collected the nuclear supernatant following 10-min centrifugation at 12,000 × g. Following pre-clearing the supernatant with streptavidin beads (New England Biolabs, Ipswich, MA, USA), we incubated the precleared lysate with biotinylated RNA-coupled magnetic beads for the pulldown assay at 4 °C. After 4 hours of hr-incubation with gentle rotation, RNA-associated proteins were collected by a magnet and eluted by using SDS lysis buffer. The associated proteins in the RNA complex were identified by mass spectrometry.

Promoter-driven luciferase reporter assay

The mutated promoter construct (pGL3-UCA1-HRE4mut) was generated by Quick Change site-directed mutagenesis (Merck KGaA, Darmstadt, Germany) using pGL3-UCA1-1411/+444 as the template and two primers for mutations (**Table S1**). The indicated cells at 5×104 /well were seeded in 24-well plates. We performed the co-transfection of proximal UCA1 promoter (-1411/+444) driven luciferase constructs harboring wildtype or mutated HRE4 (HRE4 mut) with or without the HIF1A expression vector 29 at the indicated doses in human 293T cells, followed by cell lysate

harvest for luciferase activity measurement at 48-hr post-transfection. The luciferase activity was determined using a Dual Luciferase® Reporter Assay kit (Promega, Madison, WI, USA). We used the Renilla luciferase activity to normalize Firefly luciferase activity for transfection efficiency.

Supporting Tables and Figures

Table S1 Primers and probes

Table S2 Antibody list

Fig. S1. Differential expression of *UCA1* in the indicated cancer tissues or cell lines. (**A**) A schematic diagram shows *UCA1* spliced isoforms and the primers used to quantify the expression of total *UCA*1 and those of long and short *UCA1* isoforms. The first four isoforms were experimentally validated, whereas the last one was compiled using the reported variants by NCBI. (**B**) Mean *UCA1* expression is significantly higher in tumorous tissues compared to normal tissues of BLCA (bladder cancer), BRCA (breast cancer), COAD (colon cancer), HNSC (head and neck cancer), LUSC (lung cancer), and STAD (stomach cancer) by DeepBase v3.0. (**C**) *UCA1* expression is elevated in the tumor tissues of three HPC specimens relative to their adjacent normal counterparts. ** $p<0.01$, *** $p<0.001$. (**D**) The expression of *UCA1* in the indicated HNC cell lines relative to HNOK. *** $p < 0.001$ versus HNOK. N.S., not significant.

Fig. S2. CRISPR/Cas-mediated silencing of *UCA1* expression recapitulated the depletion effect of UCA1 by using siRNA on cell proliferation, migration, and invasion. Detroit 562 cells were transduced with lentiviruses bearing sg-Ctrl or sg*UCA1* clones (clone 2 or 3). Following validation of the reduced *UCA1* expression by RT-qPCR (**A**), we assayed cell proliferation (**B**), migration (**C**), and invasion (**D**). All the experiments

were independently repeated three times and expressed as mean ± SD (N = 3). * $p < 0.05$; ** $p < 0.01$; *** $p < 0.001$ compared to sgCtrl, t-test.

Fig. S3. The expression of *UCA1* isoforms in the HNC lines and a positive correlation of *UCA1* with mesenchymal signatures in the TCGA-HNC database. (**A**) RT-qPCR analysis of two isoforms, 1.4 (white bar) and 2.3 kb (black bar), of *UCA1* in the indicated cell lines. $p < 0.05$; ** $p < 0.01$; *** $p < 0.001$ compared to NHOK, t-test. (**B**) The correlation in the mRNA expressions of UCA1 and 4 mesenchymal signature genes in the TCGA-HNC database by Pearson correlation analysis. The mesenchymal markers include *CDH2*, *SNAI1*, *TGFB,* and *FN* in TCGA-head and neck tumor tissues ($N = 519$, $\gamma = 0.12$, $p = 0.0044$).

Fig. S4. PTBP3 is identified as a *UCA1*-interacting partner. (**A**) RNA gel analysis of biotinylated *UCA1* and its antisense RNA. Markers, 5S, 18S and 28S rRNA. (**B**) Western blot analysis of PTBP3, a novel interacting partner of *UCA1*, in the protein complex pulled down by the biotinylated *UCA1* but not its antisense RNA. This result is a representative of two independent repeats. (**C**) Right, Western blot analysis of the indicated immune complexes. Left, RIP analysis showed the in vivo enrichment of *UCA1* in the PTBP3 immunocomplex relative to IgG control (N = 3). *** $p < 0.001$ compared to IgG, One sample t-test. Full-length blots are presented in Supplementary Figures S18-S19.

Fig. S5. Ectopic PTBP3 overexpression rescues *UCA1*-mediated suppression of cell migration and invasion. The effect of *PTBP3* overexpression on *UCA1*-depleted Detroit 562 cell proliferation (**A**), migration (**B**), and invasion (**C**). All the experiments were independently repeated three times and expressed as mean ± SD (N = 3). *$p < 0.05$, **

p < 0.01, *** p < 0.001 or not significant (N.S.) compared to sgCtrl + Vector cells, t-test.

Fig. S6. HIF1A potentiates *UCA1* promoter activity in a dose-dependent manner. Luciferase activity driven by the *UCA1* proximal promoter (-1411/+444) in the incremental doses of HIF1A expression (50 - 200 ng). +1 as transcription start site. ** p < 0.01, or *** p < 0.001 compared to pGL3-basic vector, t-test.

Fig. S7-S19. Full-length images for all the Western blots presented in the figures and supplementary figures. They are listed below: S7 (Fig. 3A); S8 (Fig. 3B); S9 (Fig. 3C, Top, left); S10 (Fig.3C, Top, right); S11-12 (Fig. 3C, Bottom); S13 (Fig. 3D, Right); S14 (Fig. 5A); S15 (Fig. 5F); S16 (Fig. 5G); S17 (Fig. 6A); S18 (Fig. S4B); S19 (Fig. S4C).

References

1. Larson M.H., Gilbert L.A., Wang X., Lim W.A., Weissman J.S. and Qi L.S. CRISPR interference (CRISPRi) for sequence-specific control of gene expression. Nat Protoc 8(11):2180-2196, 2013.
