# Supplementary material for: Targeting the HIF1A-UCA1-PTBP3 axis: a potential therapeutic strategy for head and neck cancer
Source: BMC Cancer. 2025 Oct 9;25:1536. doi: 10.1186/s12885-025-15020-z (PMC12512865; doi:10.1186/s12885-025-15020-z)
Supplement: Supplementary file 2 — Supplementary Material 2. Tab. S1. Primers and probes [file 12885_2025_15020_MOESM2_ESM.pdf]

Table S1. Primers and probes

Gene	Sequence
Total UCA1	F :5’- CTCTCCATTGGGTTCACCATTC -3’<br>R: 5’- GCGGCAGGTCTTAAGAGATGAG -3’
UCA1-1.4 kb	F: 5’- CACCATGCACCTTGTGACTC -3’<br>R: 5’- CGTATAGAAGACCACCTAAAC -3’
UCA1-2.3kb	F: 5’- GGAGCCAAGAAGTCTGGAG -3’<br>R: 5’- GTCATAATGGTGGAATGTCG -3’
GAPDH	F: 5’- CCCACTCCTCCACCTTTGA -3’<br>R : 5’- CCACCACCCTGTTGCTGTAG -3’
U6 snRNA	F: 5’- CGGCAGCACATATACTAAAAT<br>R: 5’- AAAATATGGAACGCTTCACGA
ACTB	F: 5’- ACTCTTCCAGCCTTCCTTCC<br>R: 5’- CTCGTCATACTCCTGCTTGC
sgUCA1	5’ GTGCATGGTGGAGAGATGAT-3’
Taqman UCA1 (Cat# 4331182)	Nucleotides 879~905 (NR_015379.3)
RNAscope™ Probe- Hs-UCA1(Cat#417521)	Nucleotides 659~2289 (NR_015379.3)
UCA1-mHRE4 -U	5’-GCCAGGTGTGGTGGC<u>AT</u>ATGCCTGTAATCCCAGC-3’
UCA1-mHRE4-L	5’-GCTGGGATTACAGGC<u>AT</u>ATGCCACCACACCTGGC-3’
