# Supplementary material for: Targeting the HIF1A-UCA1-PTBP3 axis: a potential therapeutic strategy for head and neck cancer
Source: BMC Cancer. 2025 Oct 9;25:1536. doi: 10.1186/s12885-025-15020-z (PMC12512865; doi:10.1186/s12885-025-15020-z)
Supplement: Supplementary file 3 — Supplementary Material 3. Tab. S2. Antibody list [file 12885_2025_15020_MOESM3_ESM.pdf]

Table S2. Antibody list

Antibody	Catalogue #	Company
E-cadherin (G-10)	sc-8426	Santa Cruz
N-cadherin (D4R1H)	13116	Cell Signaling Technology
Vimentin	550513	BD Pharmingen
Snail (C15D3)	3879	Cell Signaling Technology
PTBP3 (F-30)	Sc-100845	Santa Cruz Biotechnology
p-SMAD2 (138D4)	3108	Cell Signaling Technology
SMAD2 (D43B4)	5339	Cell Signaling Technology
P21	610233	BD Bioscience
Cyclin D1	Sc-717	Santa Cruz Biotechnology
Pan-CK	Sc-81714	Santa Cruz Biotechnology
Ki67	GTX16667	Genetex
Lamin B1	13435	Cell Signaling Technology
Actin	MAB1501	Millipore
TGF-β1	3709	Cell Signaling Technology
GAPDH	GTX100118	Genetex
