# Supplementary material for: Targeting the HIF1A-UCA1-PTBP3 axis: a potential therapeutic strategy for head and neck cancer
Source: BMC Cancer. 2025 Oct 9;25:1536. doi: 10.1186/s12885-025-15020-z (PMC12512865; doi:10.1186/s12885-025-15020-z)
Supplement: Supplementary file 4 — Supplementary Material 4. Fig. S1. Differential expression of UCA1 in the indicated cancer tissues or cell lines [file 12885_2025_15020_MOESM4_ESM.pdf]

Fig. S1. Differential expression of *UCA1* in the indicated cancer tissues or cell lines.

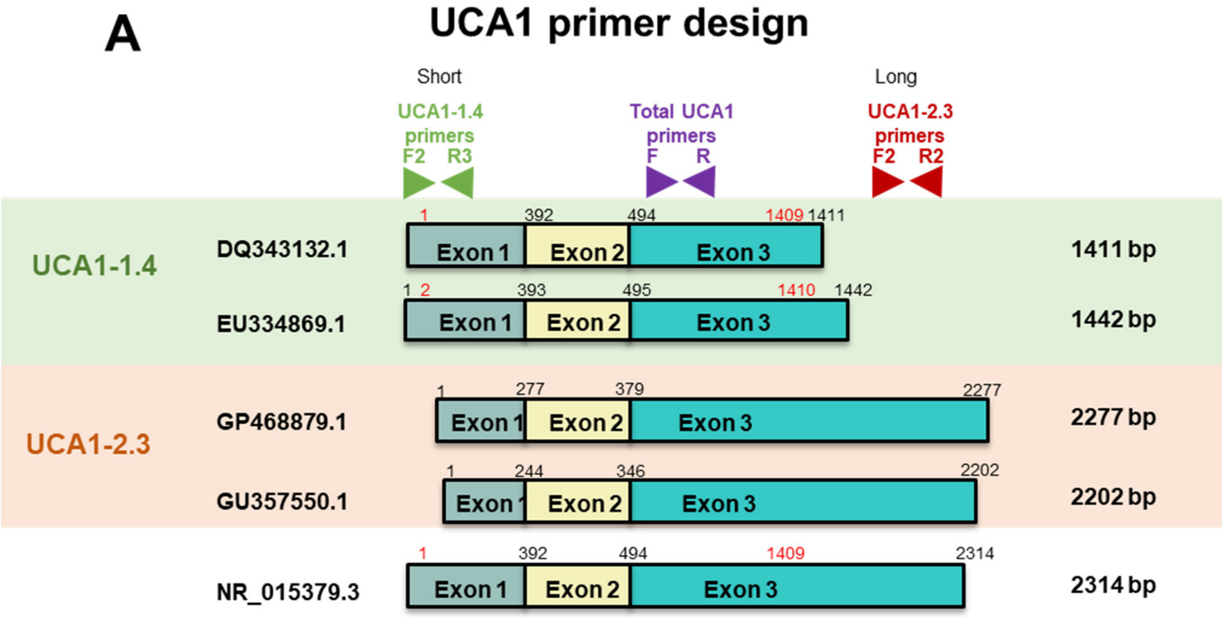

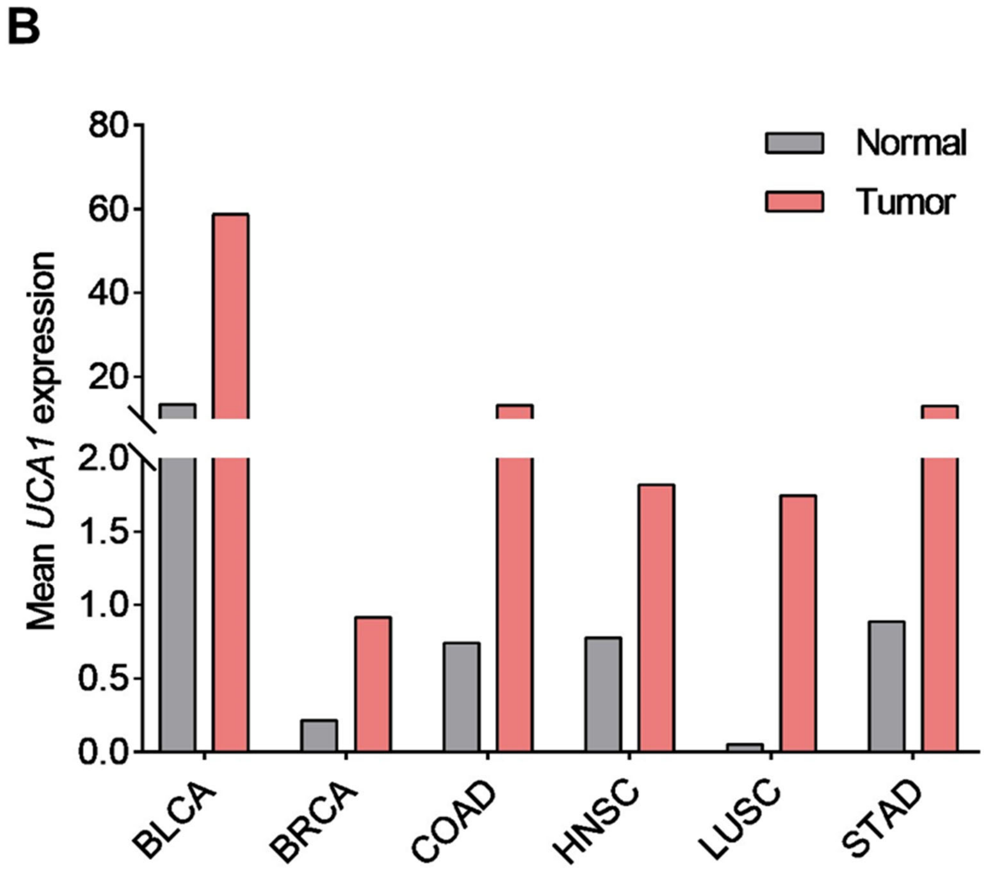

C

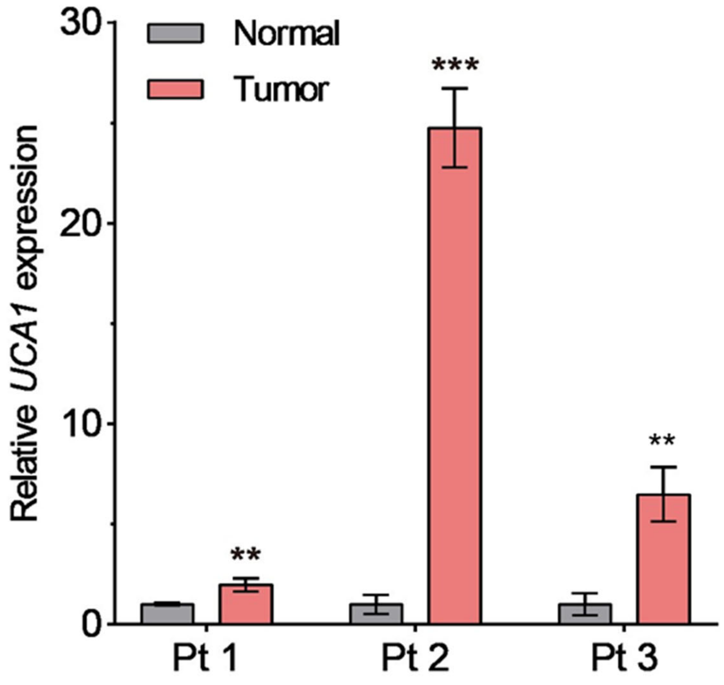

Normal
Tumor
30
20
10
0
Relative *UCA1* expression
***
**
**
Pt 1
Pt 2
Pt 3

D

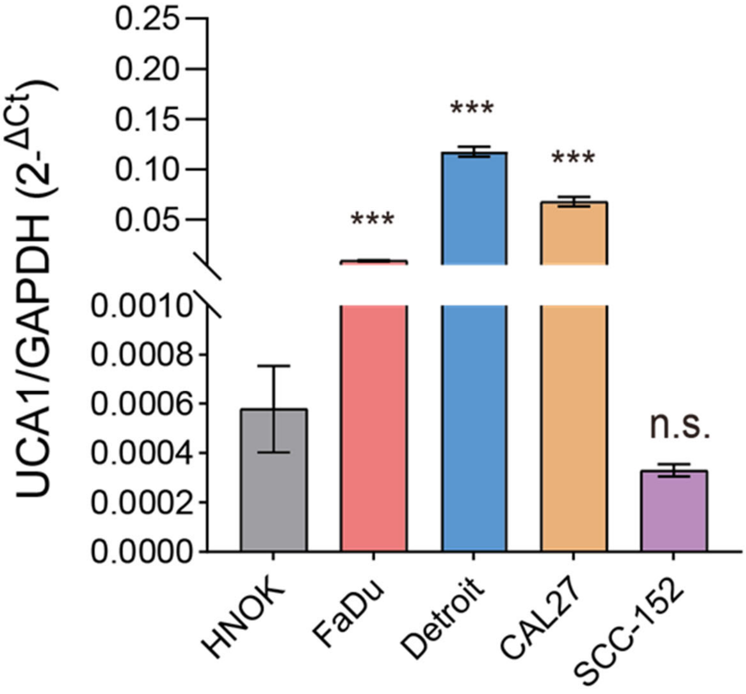

0.25
0.20
0.15
0.10
0.05
0.0010
0.0008
0.0006
0.0004
0.0002
0.0000
UCA1/GAPDH ($2^{-\Delta Ct}$)
***
***
***
n.s.
HNOK
FaDu
Detroit
CAL27
SCC-152

(A) A schematic diagram shows *UCA1* spliced isoforms and the primers used to quantify the expression of total *UCA*1 and those of long and short *UCA1* isoforms. The first four isoforms were experimentally validated, whereas the last one was compiled using the reported variants by NCBI. (B) Mean *UCA1* expression is significantly higher in tumorous tissues compared to normal tissues of BLCA (bladder cancer), BRCA (breast cancer), COAD (colon cancer), HNSC (head and neck cancer), LUSC (lung cancer), and STAD (stomach cancer) by DeepBase v3.0. (C) *UCA1* expression is elevated in the tumor tissues of three HPC specimens relative to their adjacent normal counterparts. ** $p < 0.01$, *** $p < 0.001$. (D) The expression of *UCA1* in the indicated HNC cell lines relative to HNOK. *** $p < 0.001$ versus HNOK. N.S., not significant.
