# Supplementary material for: Targeting the HIF1A-UCA1-PTBP3 axis: a potential therapeutic strategy for head and neck cancer
Source: BMC Cancer. 2025 Oct 9;25:1536. doi: 10.1186/s12885-025-15020-z (PMC12512865; doi:10.1186/s12885-025-15020-z)
Supplement: Supplementary file 5 — Supplementary Material 5. Fig. S2. CRISPR/Cas-mediated silencing of UCA1 expression recapitulated the depletion effect of UCA1 by using siRNA on cell proliferation, migration, and invasion. [file 12885_2025_15020_MOESM5_ESM.pdf]

Figure S2 CRISPR/Cas-mediated silencing of *UCA1* expression recapitulated the depletion effect of *UCA1* by using siRNA on cell proliferation, migration and invasion

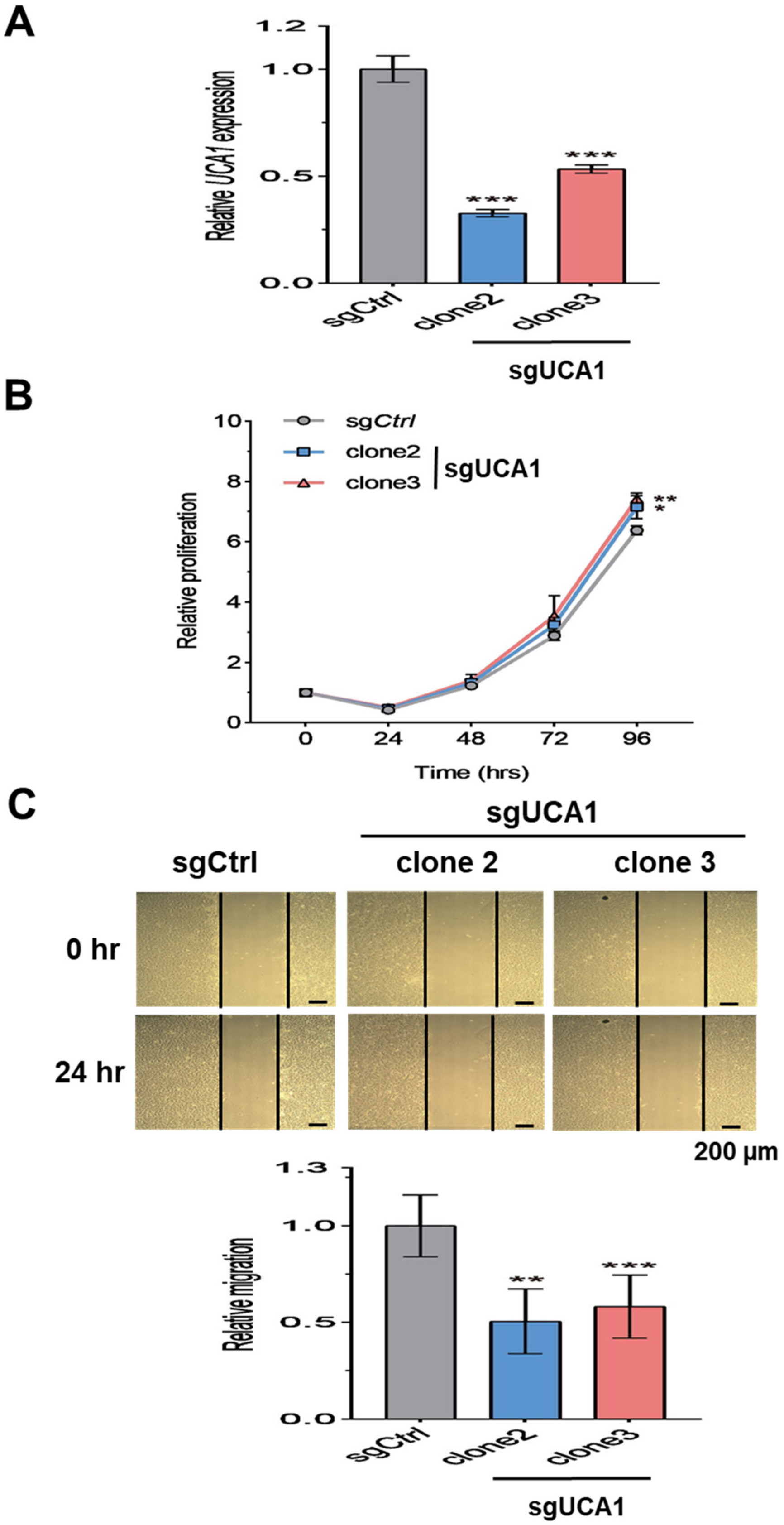

D

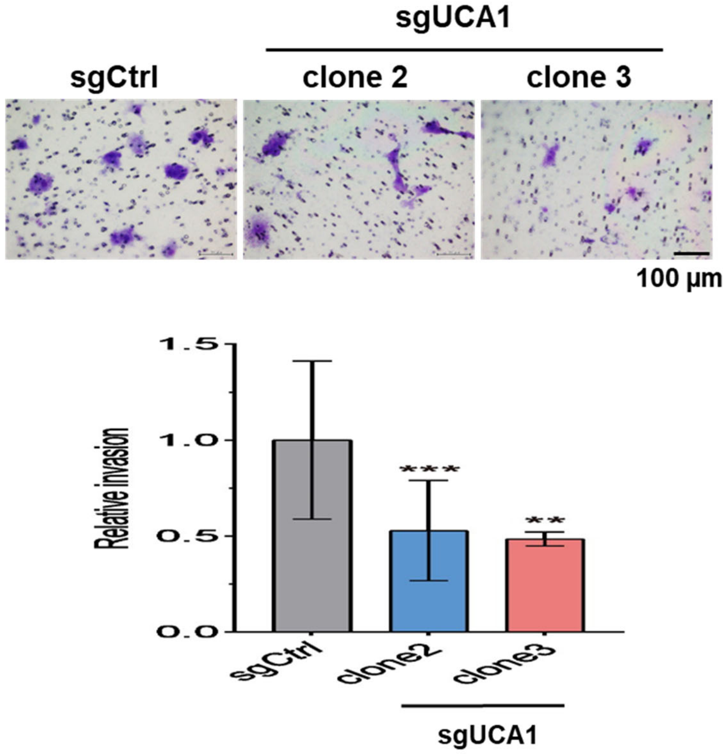

Detroit 562 cells were transduced with lentiviruses bearing sg-Ctrl or sg*UCA1* clones (clone 2 or 3). Following validation of the reduced *UCA1* expression by RT-qPCR (A), we assayed cell proliferation (B), migration (C), and invasion (D). All the experiments were independently repeated three times and expressed as mean ± SD (N = 3). * $p < 0.05$; ** $p < 0.01$; *** $p < 0.001$ compared to sgCtrl, t-test.
