# Supplementary material for: Targeting the HIF1A-UCA1-PTBP3 axis: a potential therapeutic strategy for head and neck cancer
Source: BMC Cancer. 2025 Oct 9;25:1536. doi: 10.1186/s12885-025-15020-z (PMC12512865; doi:10.1186/s12885-025-15020-z)
Supplement: Supplementary file 6 — Supplementary Material 6. Fig. S3. The expression ofUCA1 isoforms in the HNC lines and a positive correlation of UCA1 with mesenchymal signatures in the TCGA-HNC database [file 12885_2025_15020_MOESM6_ESM.pdf]

Fig. S3. The expression of *UCA1* isoforms in the HNC lines and a positive correlation of *UCA1* with mesenchymal signatures in the TCGA-HNC database.

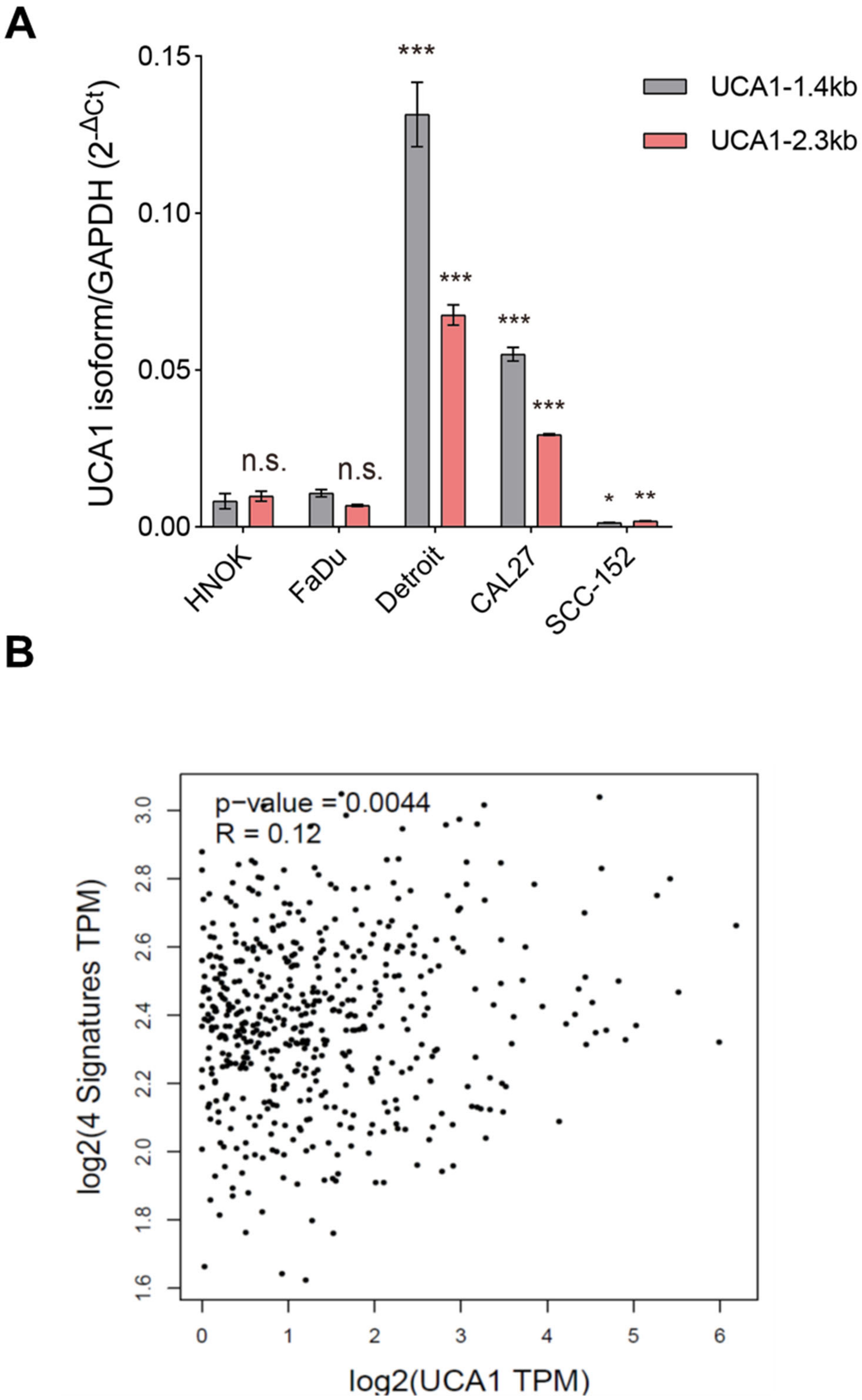

(A) RT-qPCR analysis of two isoforms, 1.4 (white bar) and 2.3 kb (black bar), of *UCA1* in the indicated cell lines. $p < 0.05$; ** $p < 0.01$; *** $p < 0.001$ compared to NHOK, t-test. (B) The correlation in the mRNA expressions of UCA1 and 4 mesenchymal signature genes in the TCGA-HNC database by Pearson correlation analysis. The mesenchymal markers include *CDH2*, *SNAI1*, *TGFB,* and *FN* in TCGA-head and neck tumor tissues ($N = 519$, $\gamma = 0.12$, $p = 0.0044$).
