# Supplementary material for: Targeting the HIF1A-UCA1-PTBP3 axis: a potential therapeutic strategy for head and neck cancer
Source: BMC Cancer. 2025 Oct 9;25:1536. doi: 10.1186/s12885-025-15020-z (PMC12512865; doi:10.1186/s12885-025-15020-z)
Supplement: Supplementary file 7 — Supplementary Material 7. Fig. S4. PTBP3 is identified as a UCA1-interacting partner [file 12885_2025_15020_MOESM7_ESM.pdf]

Fig. S4. PTBP3 is identified as a *UCA1*-interacting partner.

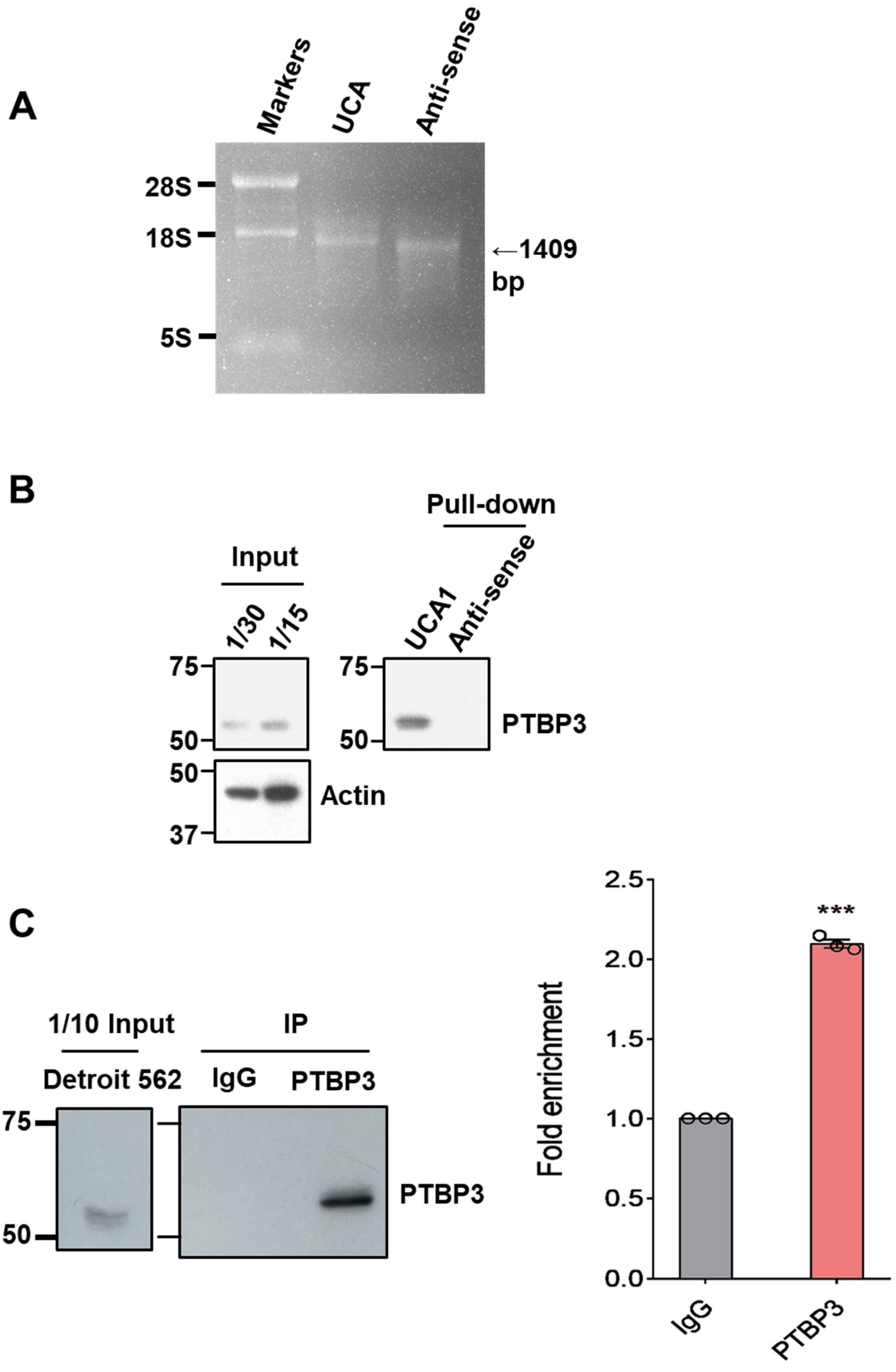

(A) RNA gel analysis of biotinylated *UCA1* and its antisense RNA. Markers, 5S, 18S and 28S rRNA. (B) Western blot analysis of PTBP3, a novel interacting partner of *UCA1*, in the protein complex pulled down by the biotinylated *UCA1* but not its antisense RNA. This result is a representative of two independent repeats. (C) Right, Western blot analysis of the indicated immune complexes. Left, RIP analysis showed the in vivo enrichment of *UCA1* in the PTBP3 immunocomplex relative to IgG control (N = 3). *** $p < 0.001$ compared to IgG, One sample t-test. Full-length blots are presented in Supplementary Figures S16-S17.
