# Supplementary material for: Targeting the HIF1A-UCA1-PTBP3 axis: a potential therapeutic strategy for head and neck cancer
Source: BMC Cancer. 2025 Oct 9;25:1536. doi: 10.1186/s12885-025-15020-z (PMC12512865; doi:10.1186/s12885-025-15020-z)
Supplement: Supplementary file 8 — Supplementary Material 8. Fig. S5. Ectopic PTBP3 overexpression rescues UCA1-mediated suppression of cell migration and invasion [file 12885_2025_15020_MOESM8_ESM.pdf]

Fig. S5. Ectopic PTBP3 overexpression rescues *UCA1*-mediated suppression of cell migration and invasion.

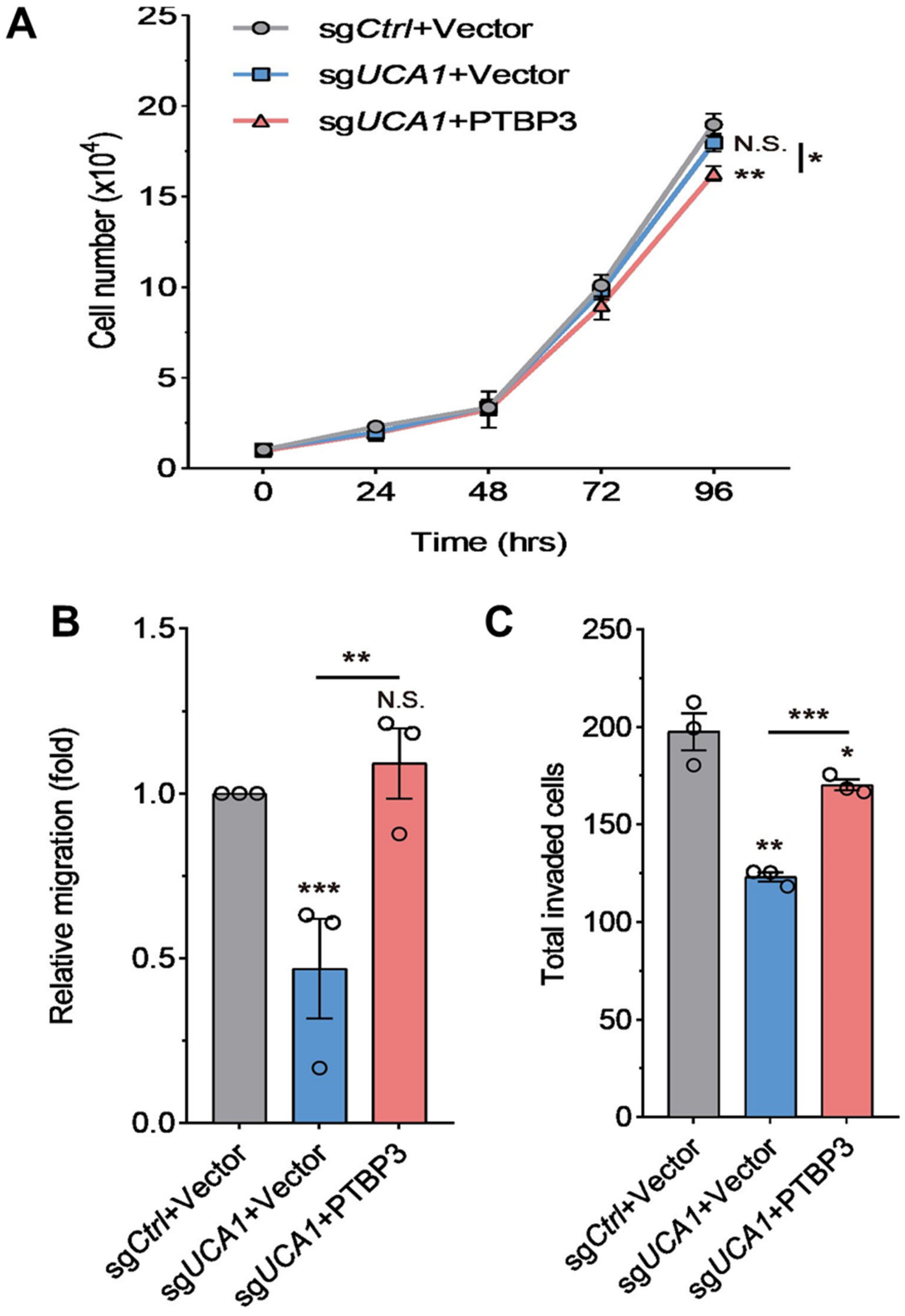

The effect of *PTBP3* overexpression on *UCA1*-depleted Detroit 562 cell proliferation (A), migration (B) and invasion (C). All the experiments were independently repeated three times

and expressed as mean ± SD (N = 3). *$p < 0.05$, ** $p < 0.01$, *** $p < 0.001$ or not significant (N.S.) compared to sgCtrl + Vector cells, t-test.
