# Supplementary material for: Targeting the HIF1A-UCA1-PTBP3 axis: a potential therapeutic strategy for head and neck cancer
Source: BMC Cancer. 2025 Oct 9;25:1536. doi: 10.1186/s12885-025-15020-z (PMC12512865; doi:10.1186/s12885-025-15020-z)
Supplement: Supplementary file 9 — Supplementary Material 9. Fig. S6. HIF1A potentiates UCA1 promoter activity in a dose-dependent manner. [file 12885_2025_15020_MOESM9_ESM.pdf]

Fig. S6. HIF1A potentiates *UCA1* promoter activity in a dose-dependent manner

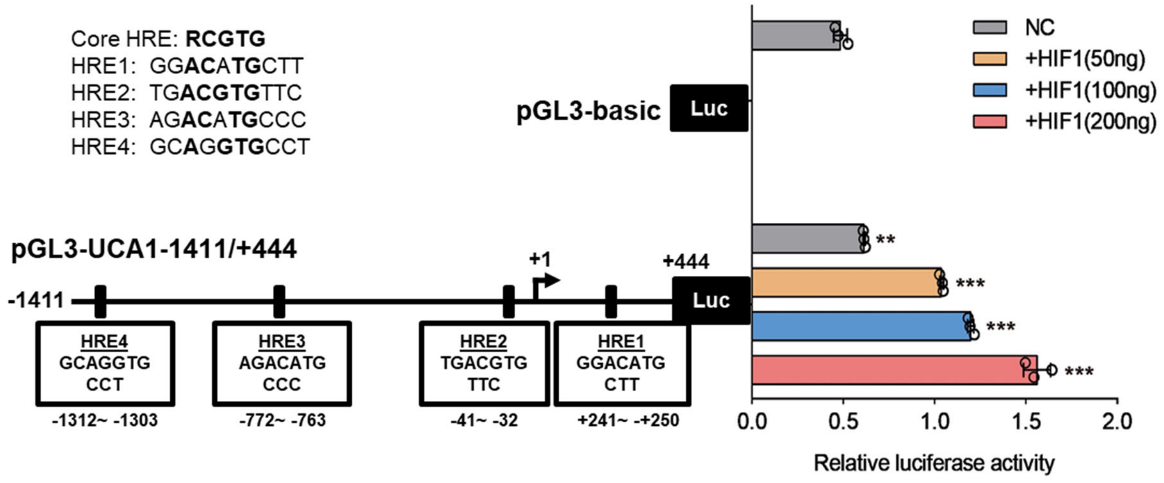

Luciferase activity driven by the *UCA1* proximal promoter (-1411/+444) in the incremental doses of HIF1A expression (50 - 200 ng). +1 as transcription start site. ** $p < 0.01$ or *** $p < 0.001$ compared to pGL3-basic vector, t-test.
