# Supplementary material for: Targeting the HIF1A-UCA1-PTBP3 axis: a potential therapeutic strategy for head and neck cancer
Source: BMC Cancer. 2025 Oct 9;25:1536. doi: 10.1186/s12885-025-15020-z (PMC12512865; doi:10.1186/s12885-025-15020-z)
Supplement: Supplementary file 10 — Supplementary Material 10. [file 12885_2025_15020_MOESM10_ESM.pdf]

Fig. S7

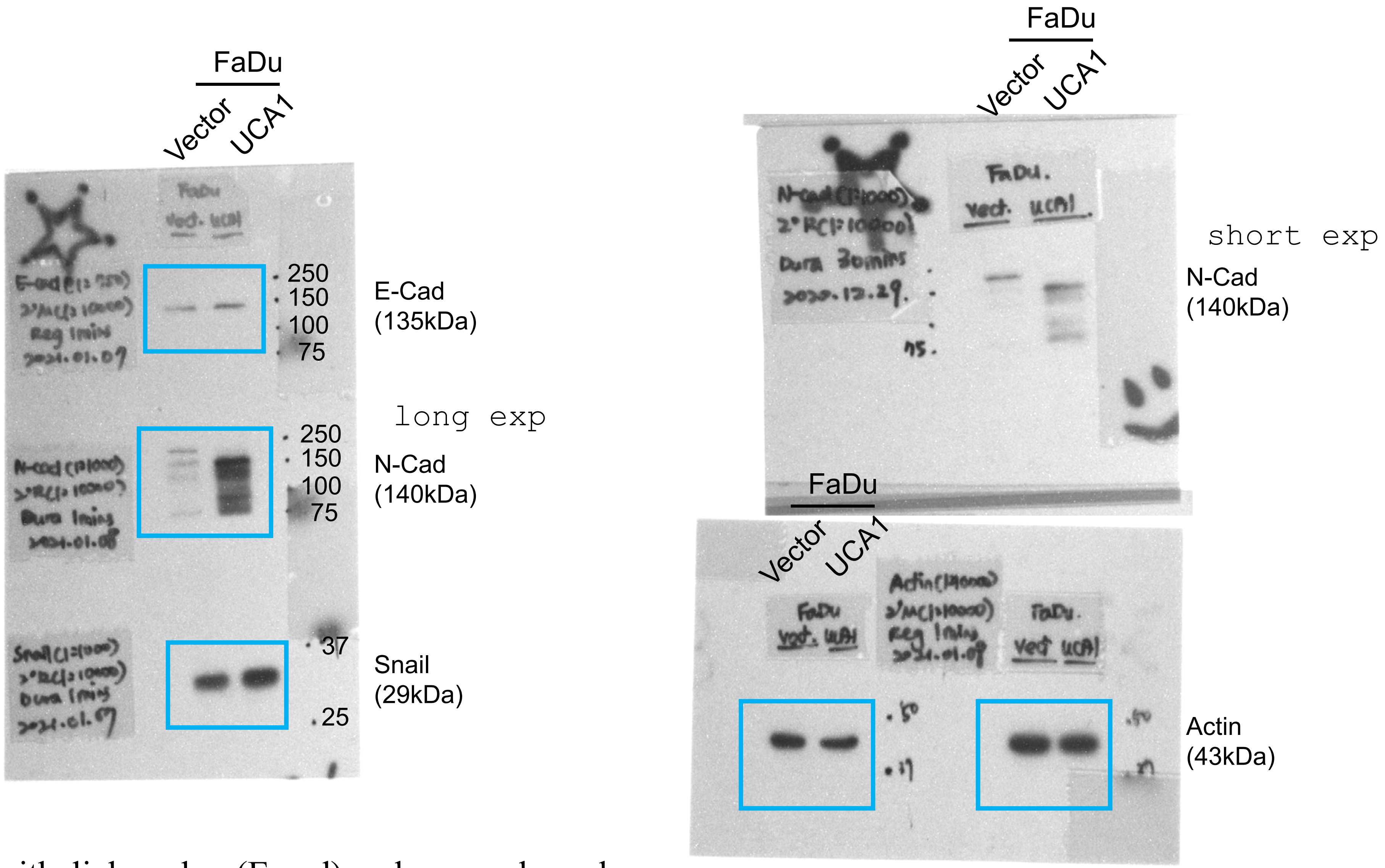

Fig. 3A Epithelial marker (E-cad) and mesenchymal markers (N-cad and snail) were measured by Western blot analysis (cut blots) in the UCA1-expressing cells

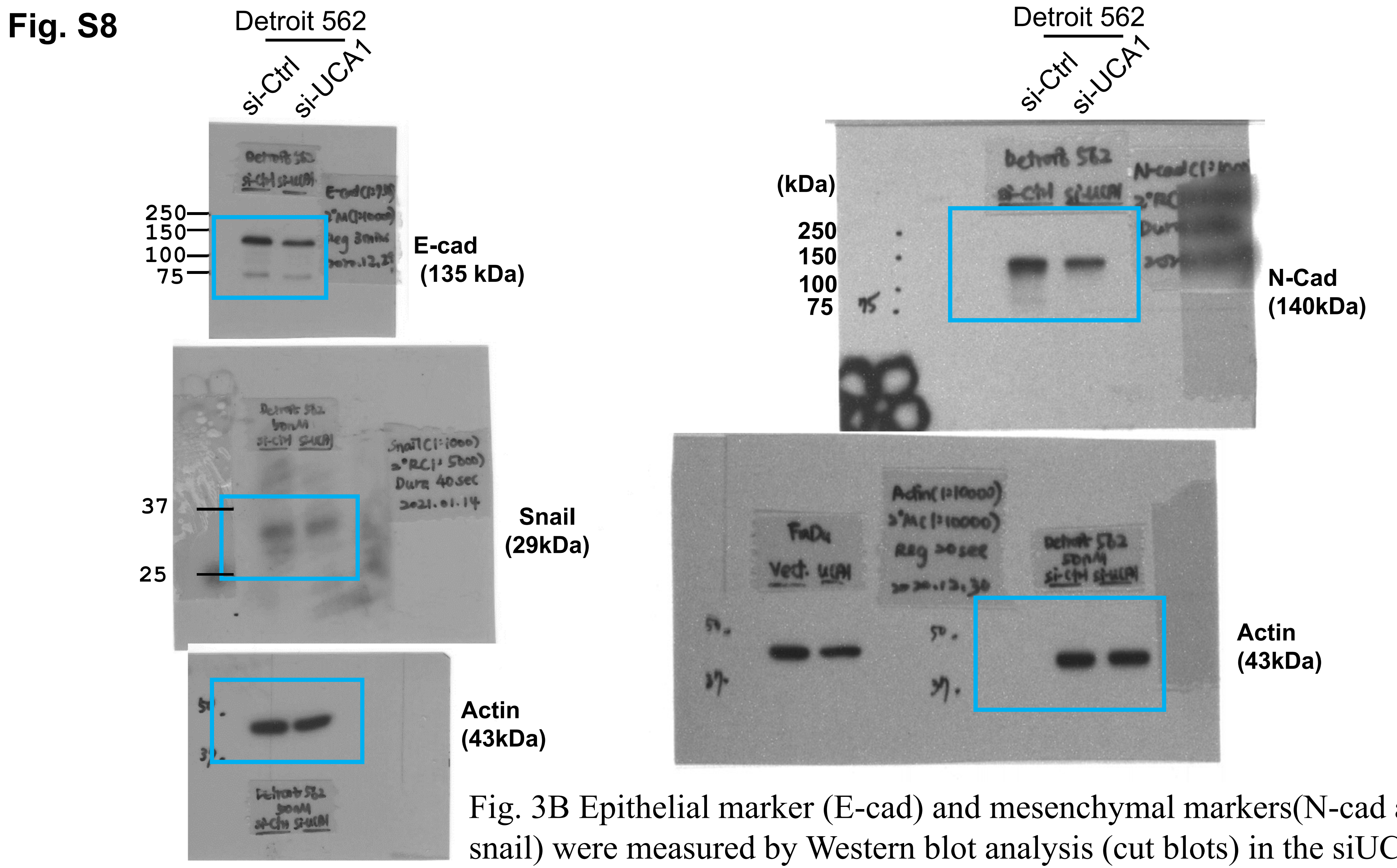

Fig. 3B Epithelial marker (E-cad) and mesenchymal markers(N-cad and snail) were measured by Western blot analysis (cut blots) in the siUCA1-expressing cells

Fig. S9

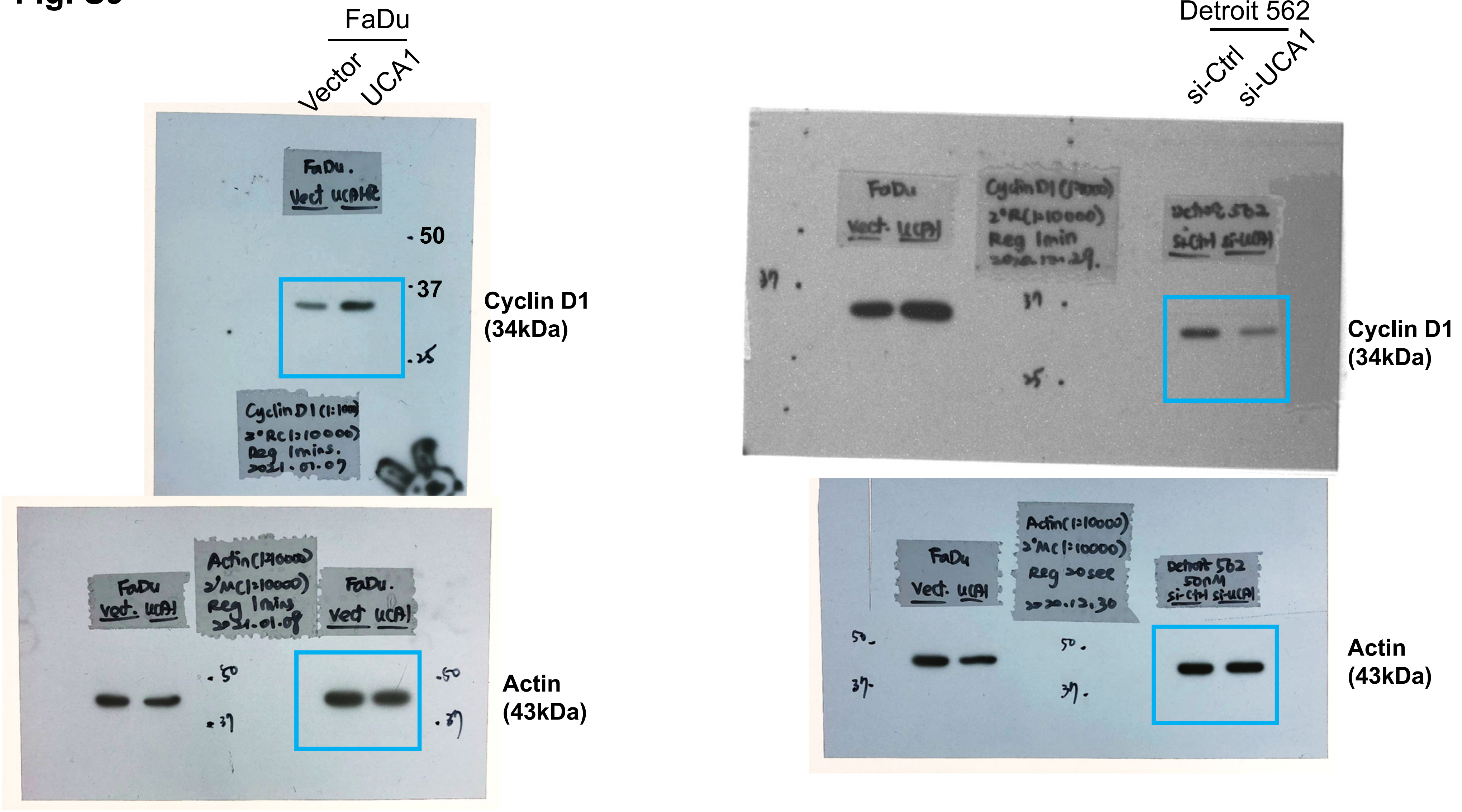

Fig. 3C Western blot analysis (cut blots) of cyclin D1 in the UCA1-manipulated FaDu or Detroit 562 lines

Fig. S10

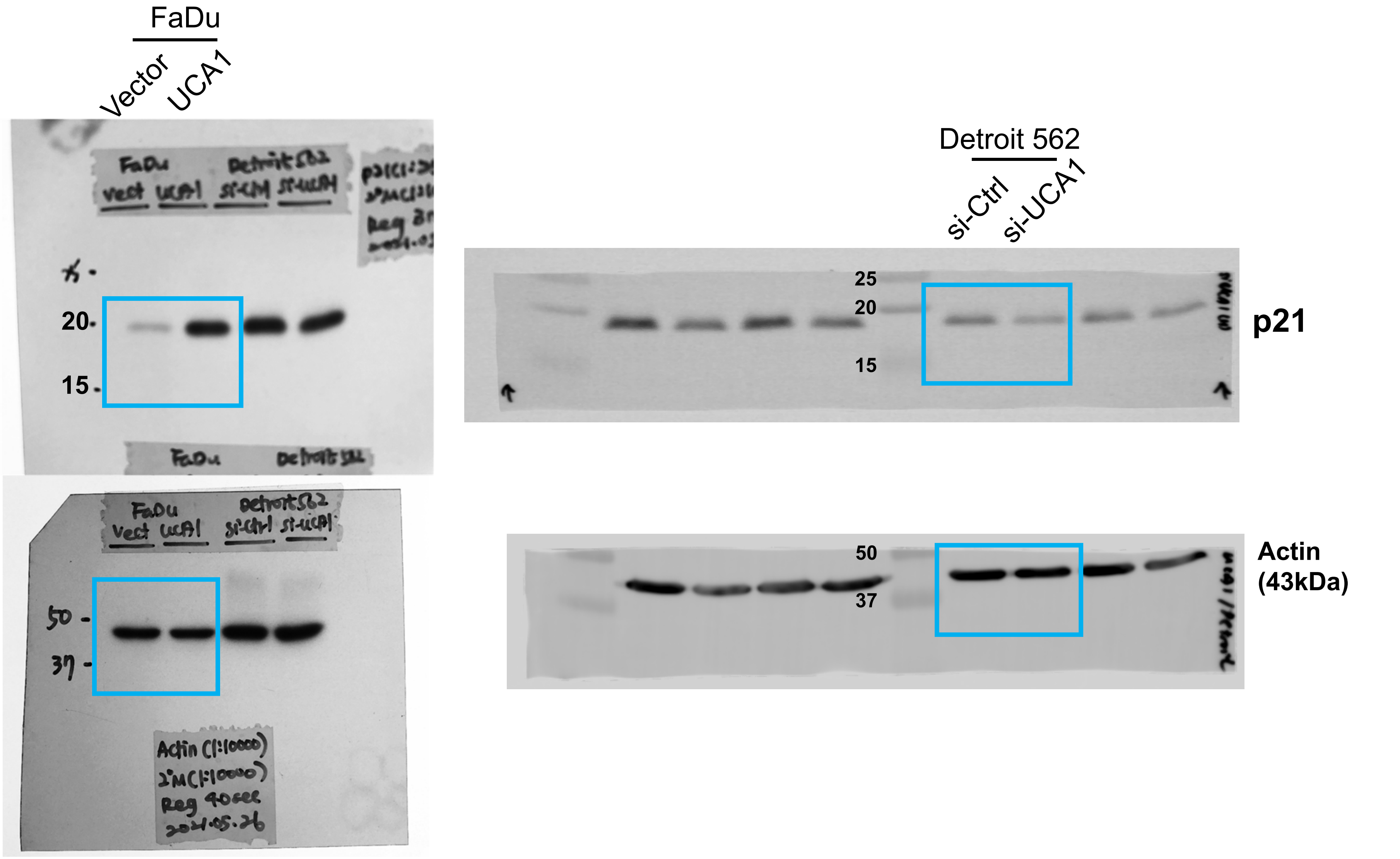

Fig. 3C Western blot analysis (cut blots) of p21 in the UCA1-manipulated FaDu or Detroit 562 lines

Fig. S11

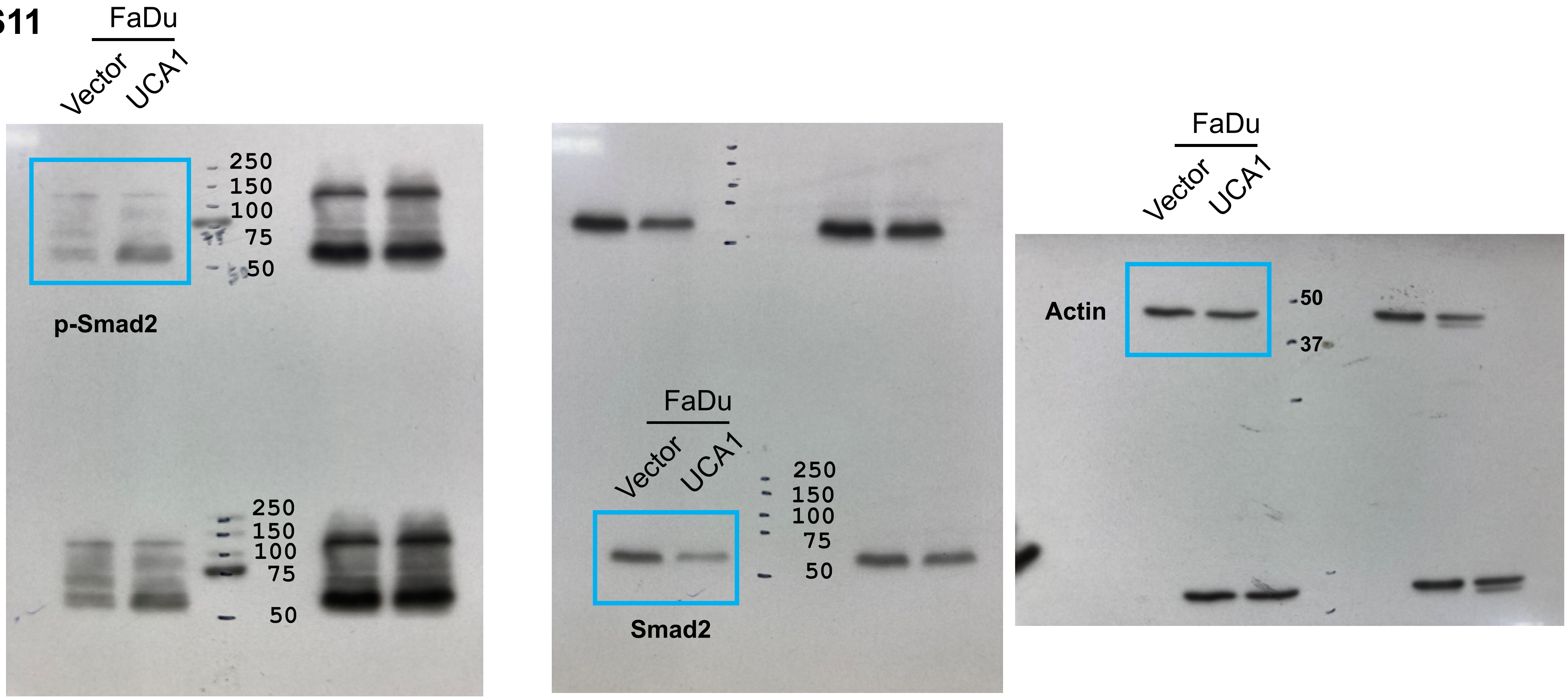

Fig. 3C The activating phosphorylation at Ser465/467 of Smad2 (p-Smad2) and its total level in UCA1-expressing FaDu line by Western blot analysis (cut blots)

Fig. S12

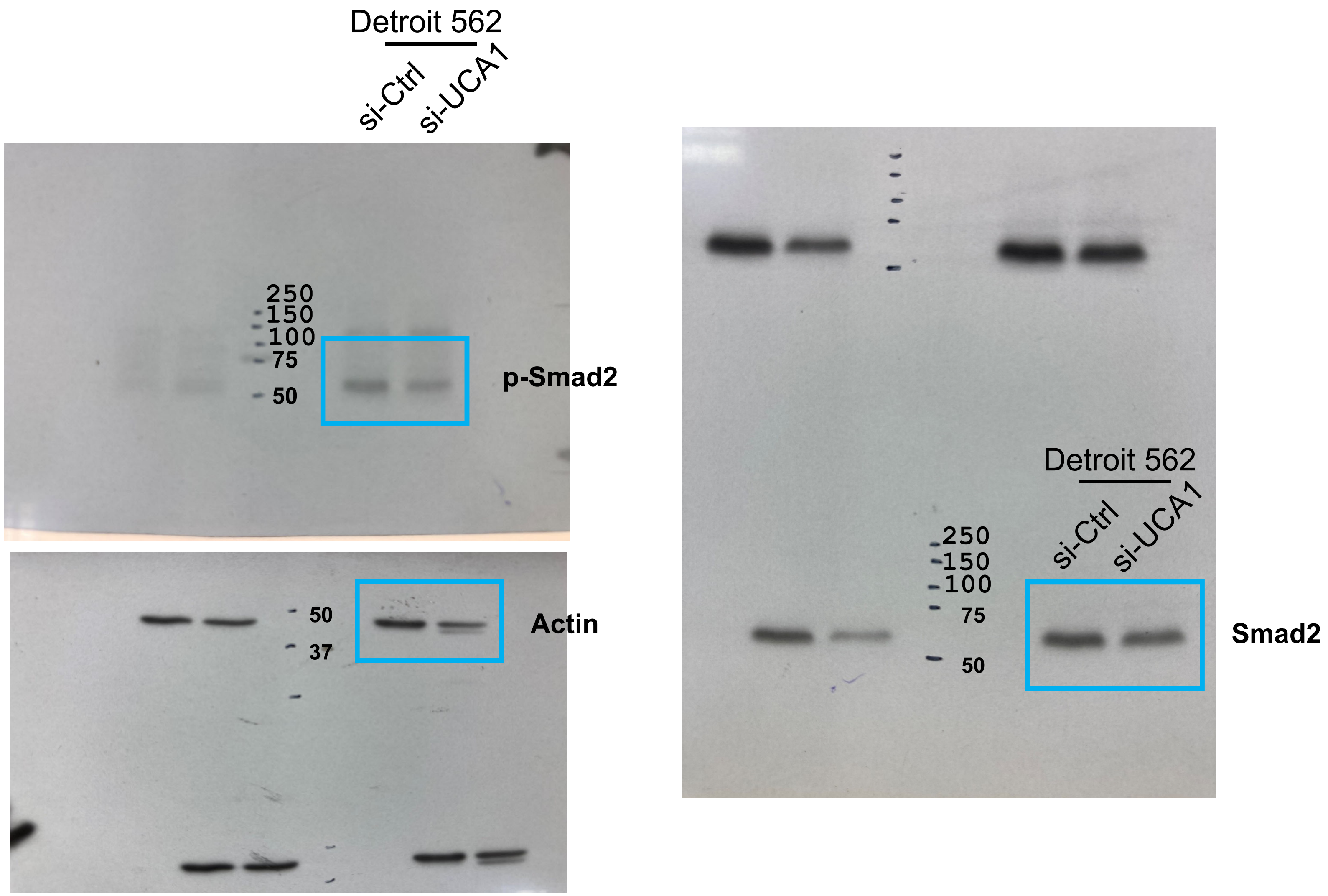

Fig. 3C The activating phosphorylation at Ser465/467 of Smad2 (p-Smad2) and its total level by Western blot analysis (cut blots)in the si-UCA1-expressing Detroit 562 cells

Fig. S13

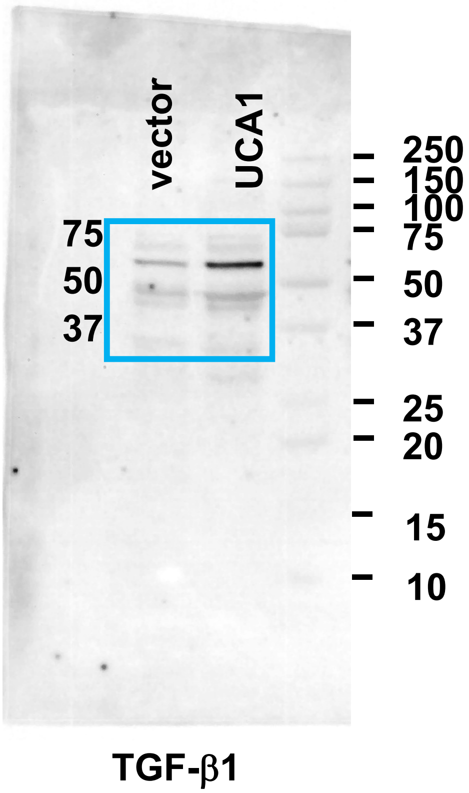

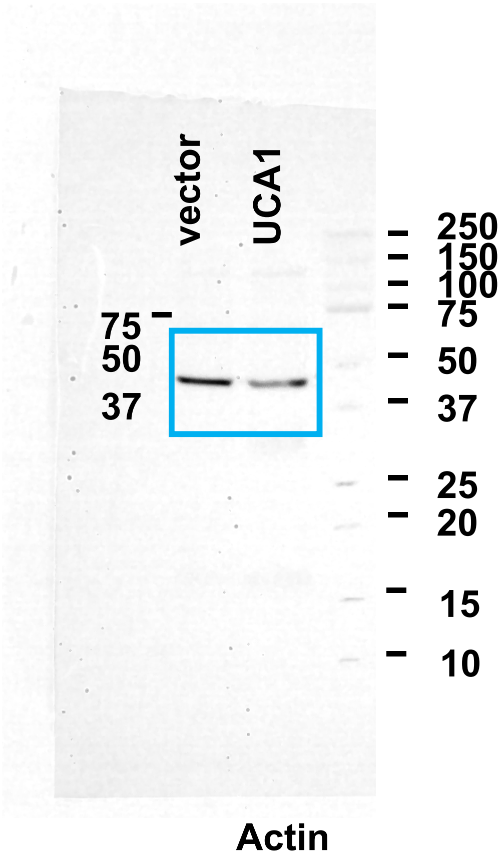

Fig. 3D (Right) Western blot analysis of TGFB1 in UCA1-expressing FaDu line (aken by using e-Blot Touch Imager, a new generation of WB imaging technology)

Fig. S14

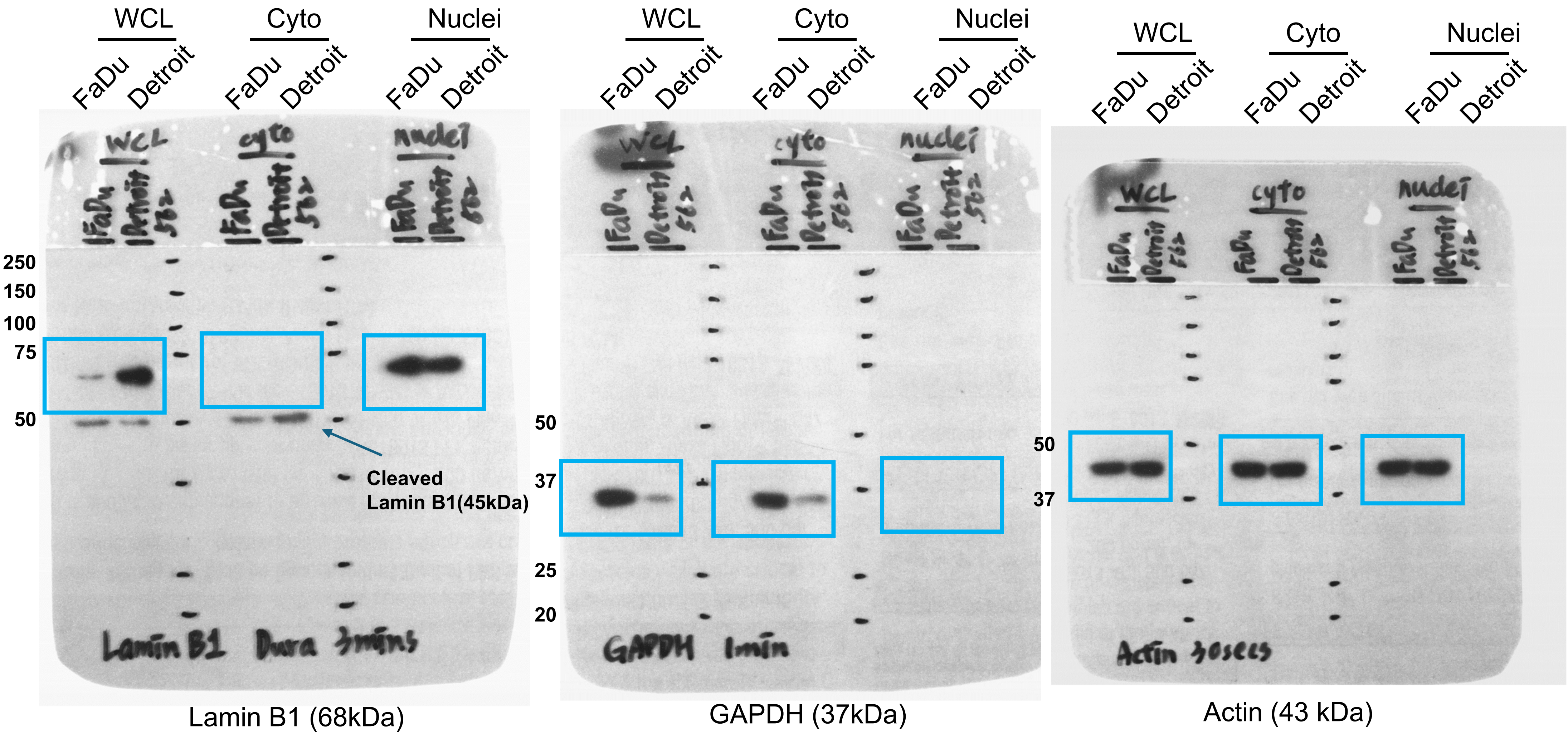

Fig.5A Western blot analysis of nuclear (lamin B1) and cytoplasmic (GAPDH) in the whole cell lysates (WCL) and subcellular fractions

Fig. S15

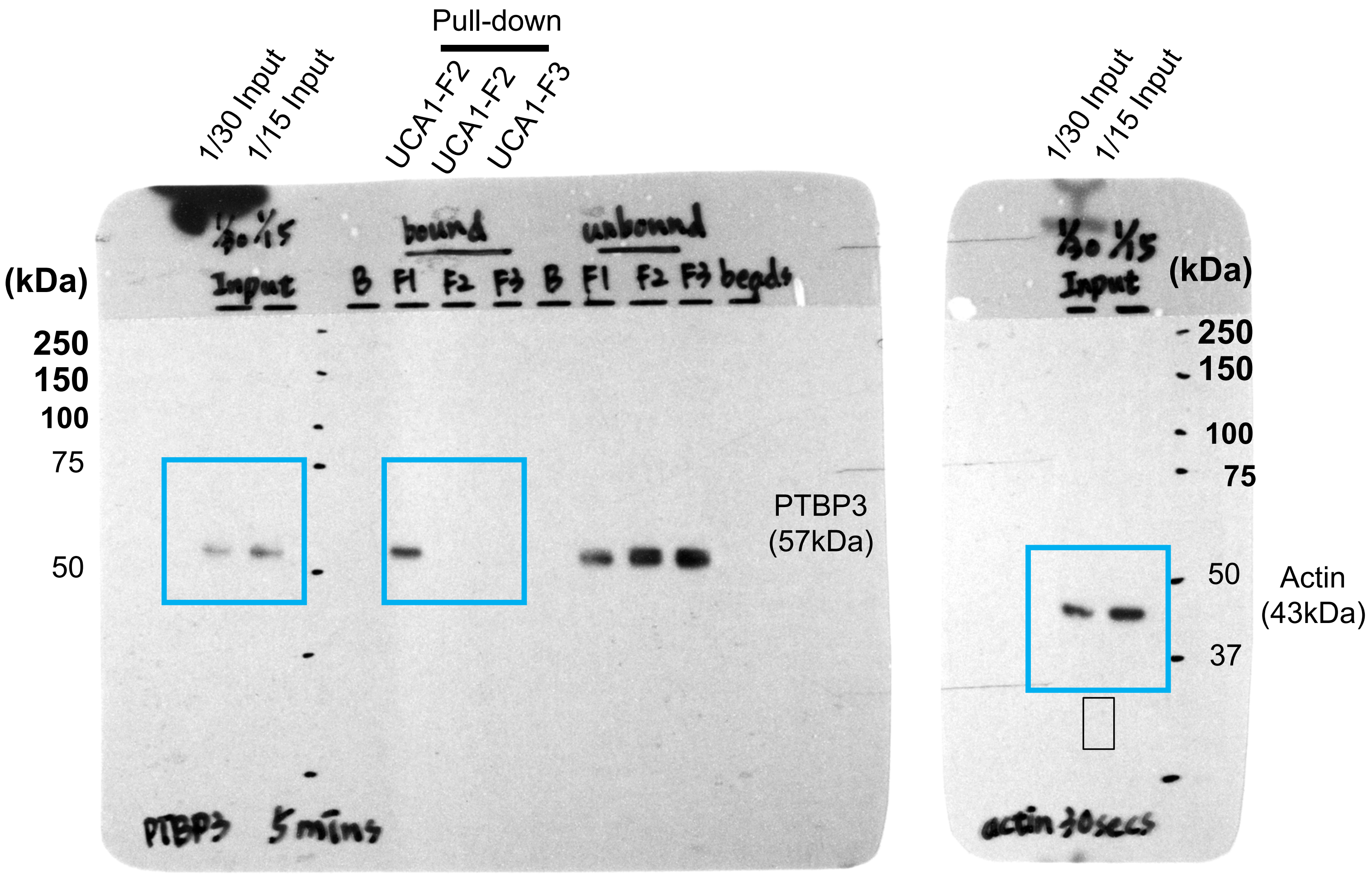

Fig. 5F Western blot analysis of PTBP3 differentially bound to 3 segments, F1-F3 of the 1.4 kb UCA1 following RNA pulldown assays

Fig. S16

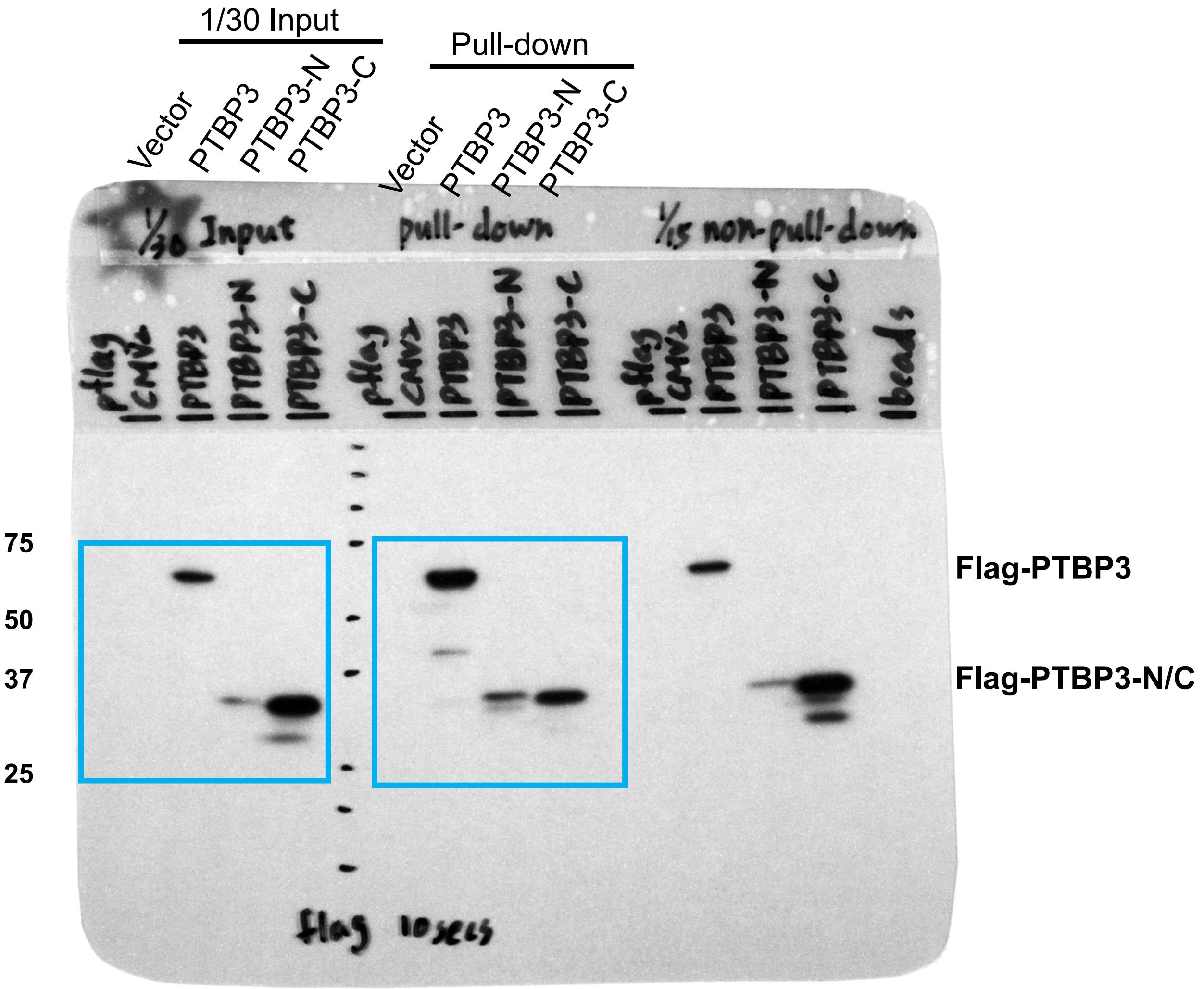

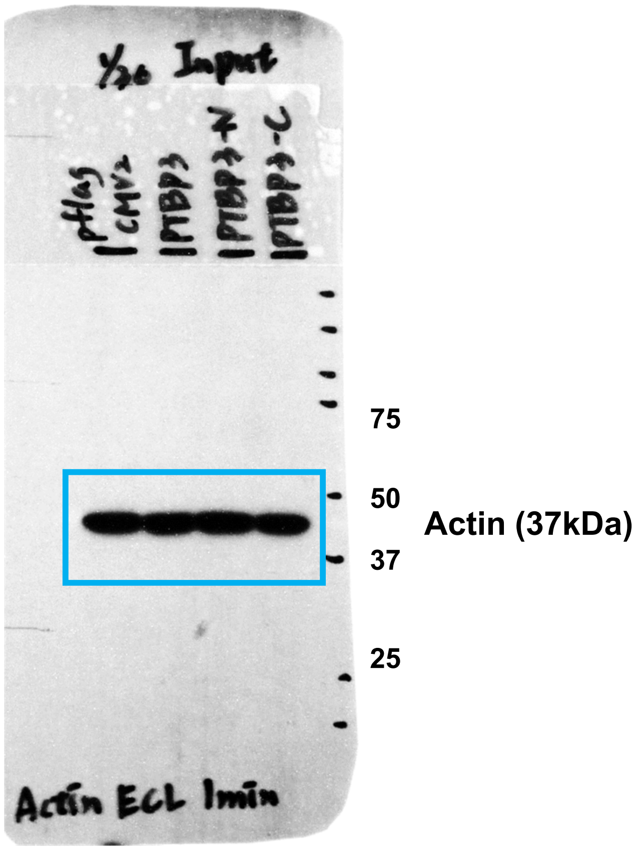

Fig. 5G Western blot analysis of the UCA1 binding to the full length of PTBP3 and its truncated forms, PTBP3-N and PTBP3-C.

Fig. S17

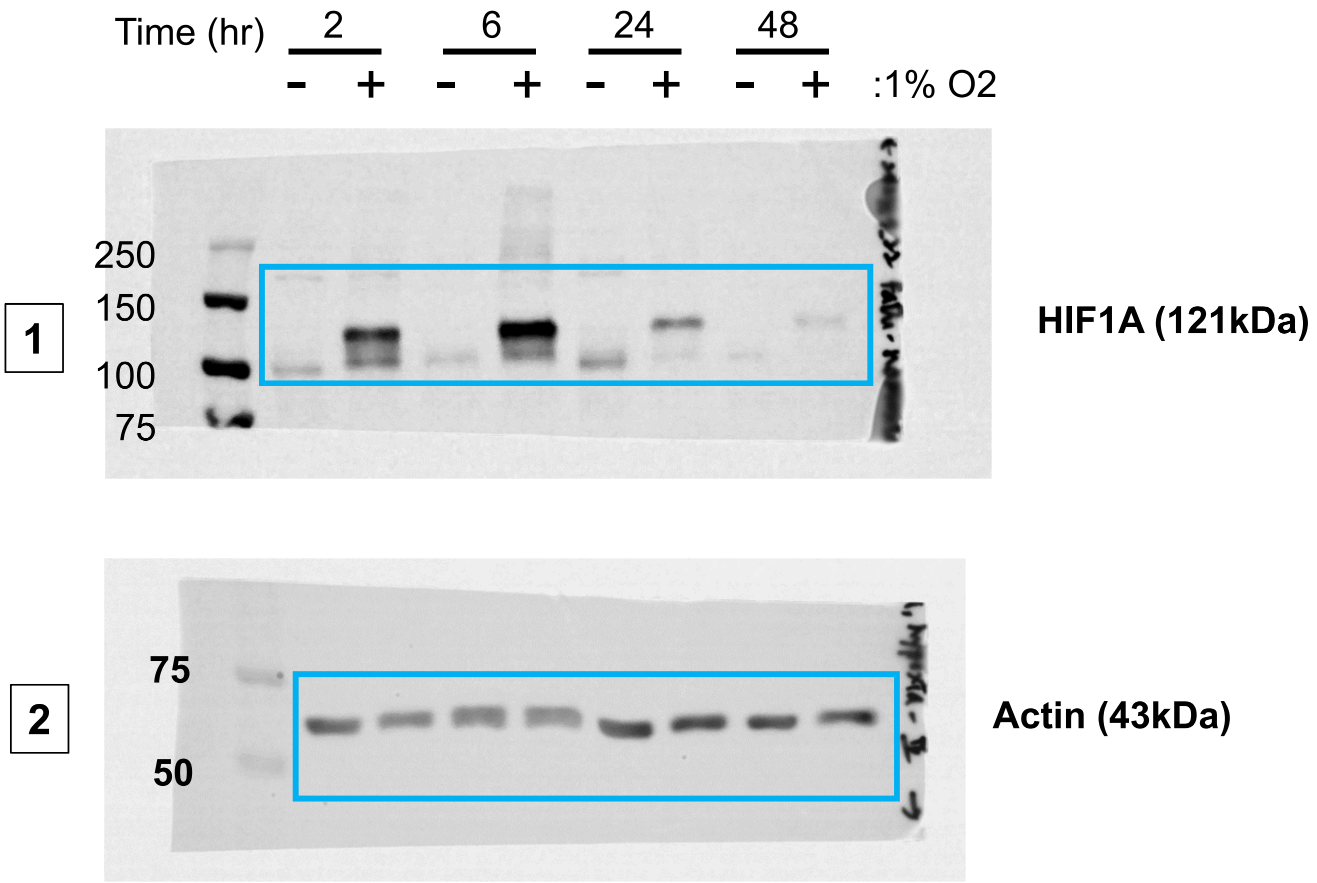

Fig. 6A HIF1A expression by Western blot analysis (cut blots) in the FaDu cells under normoxia or hypoxia

Fig. S18

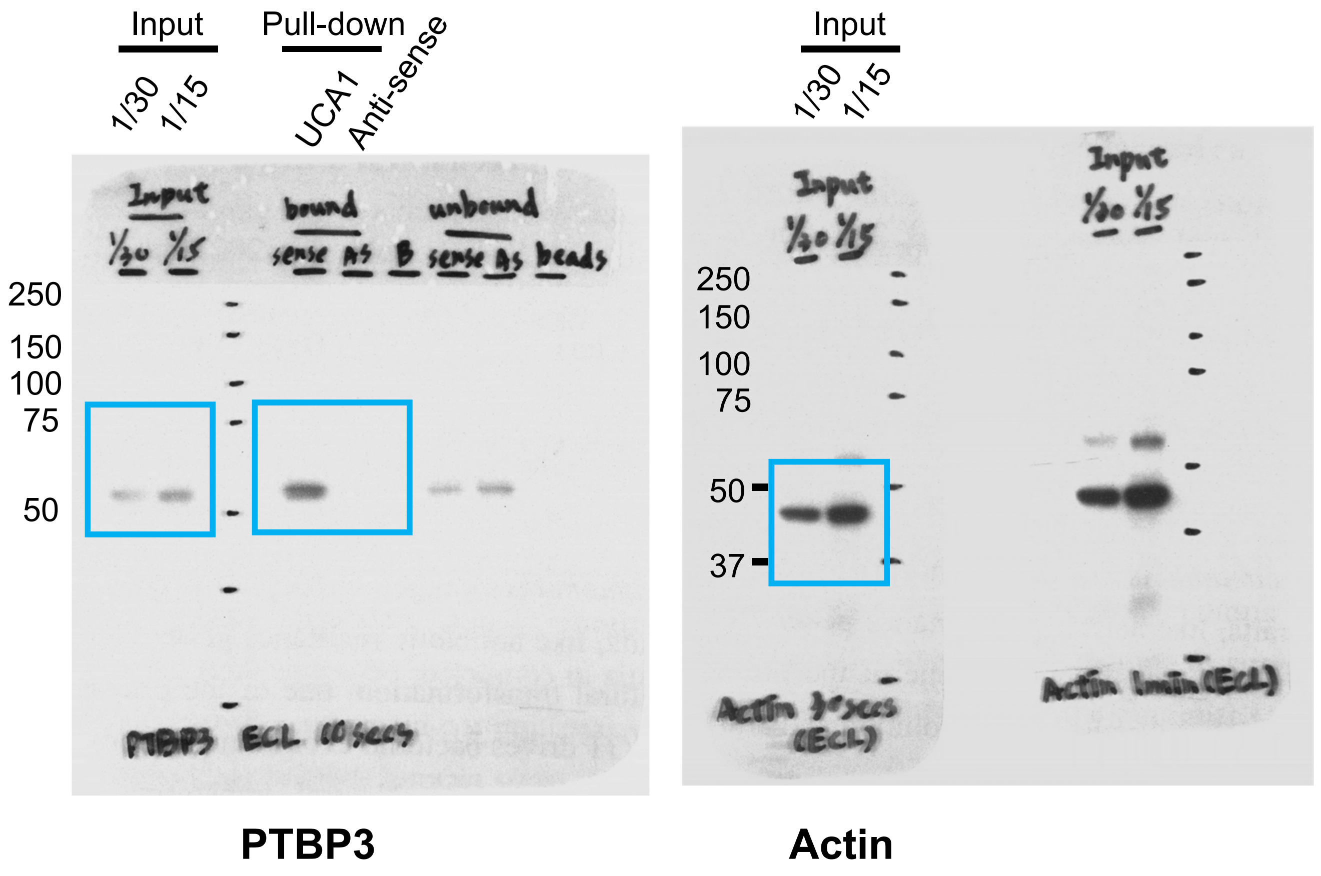

Fig. S4B Western blot analysis of PTBP3in the protein complex pulled down by the biotinylated UCA1 but not its antisense RNA

Fig. S19

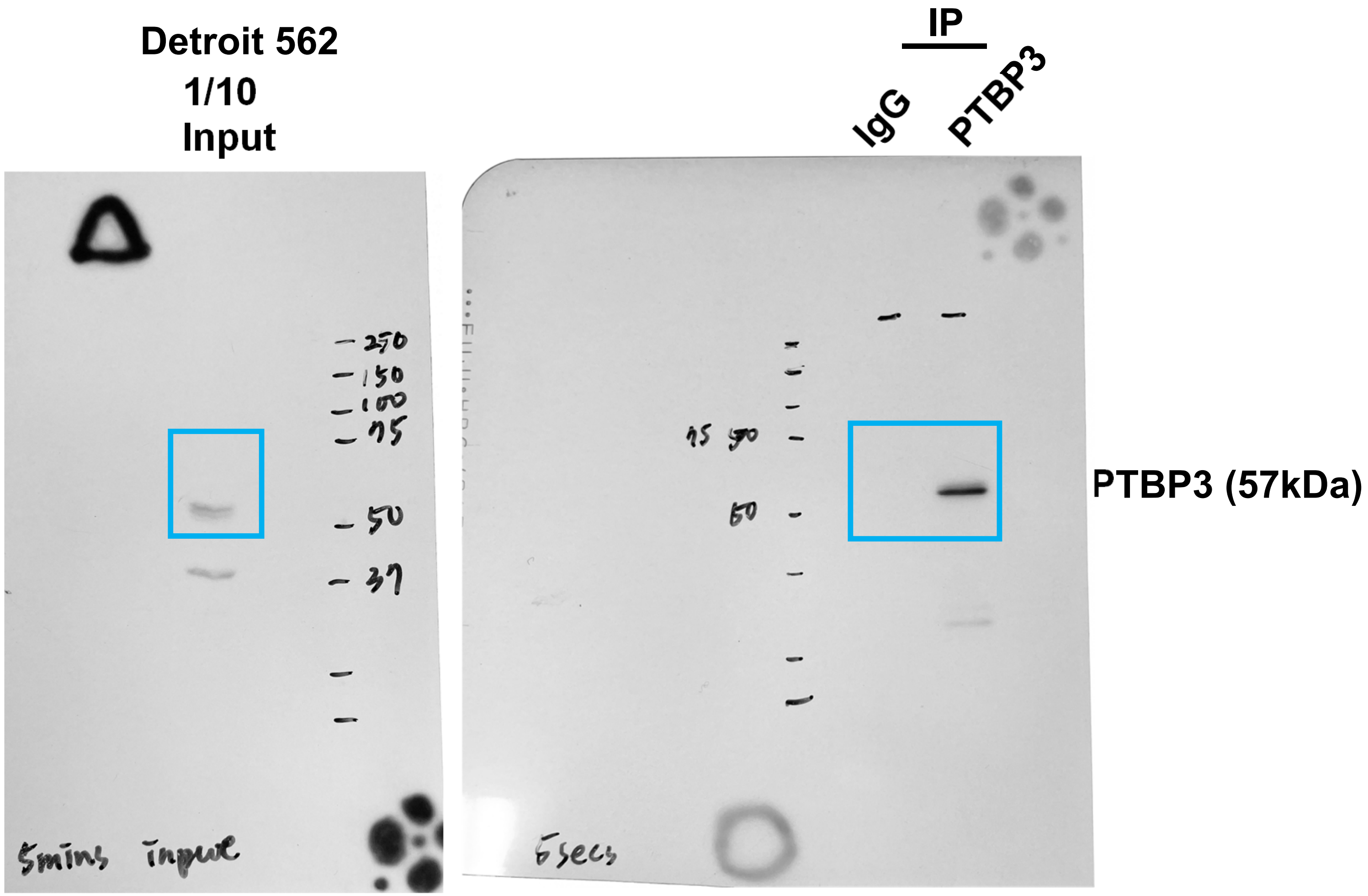

Fig. S4C Western blot analysis of the indicated immune complexes following RIP assay
